# Interconnected roles of mitochondrial carrier proteins ANT, P_i_T, and UCPs in proton transport

**DOI:** 10.3389/fmolb.2025.1650261

**Published:** 2025-11-24

**Authors:** Marzieh Tabefam, Heidi K. Swanson, Matthew D. Smith, Masoud Jelokhani-Niaraki

**Affiliations:** 1 Department of Chemistry and Biochemistry, Wilfrid Laurier University, Waterloo, ON, Canada; 2 Department of Biology, Wilfrid Laurier University, Waterloo, ON, Canada

**Keywords:** membrane protein, membrane transport, mitochondrial inner membrane, mitochondrial carrier proteins, proton transport, adenine nucleotide translocase, phosphate translocase, uncoupling proteins

## Abstract

**Introduction:**

Adenine nucleotide translocase (ANT), phosphate translocase (P_i_T), and uncoupling proteins (UCPs), all integral to oxidative phosphorylation, are among the carrier proteins of the mitochondrial inner membrane (MIM). While traditionally thought to function as monomers, their close proximity within the densely packed MIM suggests potential mutual interactions and formation of homo- and/or hetero-oligomers, the physiological implications of which are yet to be understood.

**Methods:**

We investigated the conformations and proton transport activity of ANT1, P_i_T, UCP2 and UCP4 individually and in combination, to explore the possibility of hetero-oligomerization and functionally relevant interactions among the proteins. Monomeric proteins were reconstituted, individually and/or in combination, into model lipid membranes and the conformation, oligomeric state, and proton transport activities were assessed using biophysical approaches.

**Results:**

UCP2 and UCP4 spontaneously assembled into functional tetramers, whereas ANT1 and P_i_T predominantly remained monomeric. The presence of cardiolipin in lipid membranes affected ANT1 oligomerization but had no influence on UCPs or P_i_T, suggesting that homotetramerization may be a characteristic of only a subset of mitochondrial carriers. Nevertheless, binary and ternary combinations of the proteins formed heterotetramers capable of proton transport. The UCP2-ANT1 combination showed significant proton transport, whereas proton transport by the UCP4-P_i_T combination was substantially lower, highlighting the specificity of interactions. Proton transport was differentially activated by free fatty acids; oleic acid was a better activator than palmitic acid. Inhibitory effects of purine nucleotides also varied across different protein combinations.

**Discussion:**

Collectively, our findings emphasize how interactions among these four mitochondrial carrier proteins may affect proton transport across the MIM and influence mitochondrial bioenergetics.

## 1 Introduction

The mitochondrial inner membrane (MIM) contains a diverse group of solute carriers known as the mitochondrial carrier (MC) protein family or solute carrier family 25 (SLC25). These relatively small transport proteins (∼30–40 kDa) are crucial for eukaryotic metabolism; they mediate the exchange of solutes between the mitochondrial matrix and cytosol, and link mitochondrial functions with those of the rest of the cell (Kunji and Ruprecht, 2020; Busch et al., 2023). The human genome encodes 53 different MC protein family members that transport a wide array of substrates of variable size including protons, inorganic ions, co-factors, nucleotides, amino acids, fatty acids, and di- and tri-carboxylates. Structurally, most MC protein family members share a common overall fold and membrane topology, and feature three repeated domains. Each domain is approximately 100 residues in length, comprising two transmembrane α-helices, with both the N- and C- termini oriented toward the intermembrane space. Each repeat of two α-helices is connected by a short extra-membranous amphipathic helix that is oriented parallel to the lipid bilayer in the matrix (Busch et al., 2023). A single conserved substrate or inhibitor binding site is thought to reside at the centre of the transporter cavity, with the central pore being alternately closed by salt bridge networks near the matrix side (m-gate) or the cytoplasmic side (c-gate) that faces the intermembrane space. This structural knowledge is largely based on the high-resolution X-ray structure of bovine adenine nucleotide translocase 1 (ANT1) in complex with carboxyatractyloside (CATR) (2.2 Å resolution, PDB code:1OKC) (Pebay-Peyroula et al., 2003).

**FIGURE 1:**
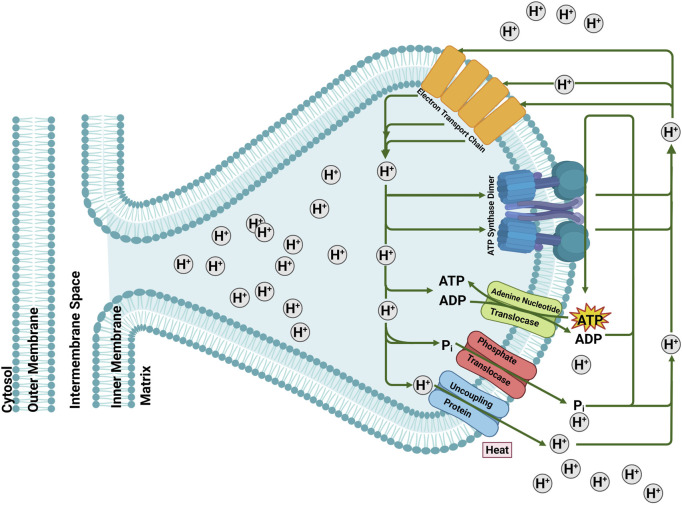
The role of mitochondrial carriers Adenine Nucleotide Translocase (ANT), Phosphate Translocase (P_i_T) and Uncoupling Protein (UCP) in mitochondrial energy-transfer reactions. The electrochemical membrane potential generated by the electron transport system is either used for ATP synthesis, electrophoretic ATP export, ΔpH-driven phosphate imports or dissipated by the uncoupling protein by regulated proton leak. Figure generated with BioRender.com.

MC protein family members are classified into two main functional groups: those associated with carbon and hydrogen metabolism and those involved in energy storage, transfer, and metabolic regulation. The latter group includes three carriers responsible for the transport of ADP and ATP, inorganic phosphate (P_i_), and protons (H^+^). ANT [also known as ADP/ATP carrier or AAC] and phosphate translocase (P_i_T) [also known as phosphate carrier (P_i_C)] are central to the energy shuttle within eukaryotic cells (Figure 1) (Krämer, 1996; Klingenberg, 2008). ANTs (ANT 1-4) (Stepien et al., 1992; Doerner et al., 1997; Lim et al., 2015), the most abundant proteins in mitochondria, account for up to 1% of total mitochondrial protein content and facilitate 1:1 exchange of ADP into and ATP out of the mitochondrial matrix. P_i_Ts (P_i_T-A and P_i_T-B) (Dolce et al., 1994; Dolce et al., 1996; Fiermonte et al., 1998), in symport with H^+^, transport P_i_ from the intermembrane space into the matrix (Krämer, 1996). Together, ANT and P_i_T supply substrates (ADP and P_i_) to ATP synthase, which generates ATP, and thus play a central role in facilitating the exchange of essential energy metabolites between mitochondria and cytosol, ensuring the continuous flow of energy to sustain cellular functions and metabolic homeostasis. The third type of MC involved in energy metabolism is a group of proteins called Uncoupling Proteins (UCPs). UCPs act as H^+^ transporters, dissipating the H^+^ gradient across the MIM established by the electron transport chain, thereby diminishing the proton motive force and reducing ATP production by ATP synthase (Figure 1) (Jacobsson et al., 1985; Rousset et al., 2004; Ardalan et al., 2022; Hoang et al., 2015; Hirschenson et al., 2022). Therefore, oxidative phosphorylation relies in part on the collective activities of ANT, P_i_T and UCP; higher UCP activity (and therefore lower proton motive force) can be thought of as offsetting the activities of ANT and P_i_T (Figure 1) (Boss et al., 1998). There are five tissue-specific UCPs in mammals (UCP1-5) (Rousset et al., 2004); UCP1, also known as thermogenin, dissipates the energy stored in the H^+^ gradient as heat (Jacobsson et al., 1985). This regulated proton leak by UCPs is activated by long-chain fatty acids and inhibited by purine nucleotide di- or tri-phosphates (i.e., GTP, GDP, ATP, and ADP) (Krauss et al., 2005). ANT and P_i_T are also known to be involved in fatty acid-mediated proton leak across the MIM, contributing to basal proton leak linked to basal metabolic rate (Engstová et al., 2001; Brand et al., 2005).

Oligomerization, where proteins form quaternary structures with multiple copies of themselves or with heterologous partners, facilitates diverse functional roles and is a well-established phenomenon in several protein families. For instance, examples of drug transporters of ATP-binding cassette and solute carrier (SLC) superfamilies are known to form functional homo- and hetero-oligomers that influence their expression level, transport function, and regulatory mechanisms (Ni and Hong, 2024). Mutual interactions caused by protein-protein and/or protein-lipid interactions for MC protein family members, particularly those involved in proton transport (such as ANT, P_i_T and UCP), remain less understood. So far, there is no consensus on the functional oligomeric forms of these proteins. It has been suggested that ANT forms either a monomer (Kunji and Ruprecht, 2020) or a homodimer (Nury et al., 2005) or both (Ardalan et al., 2021a). ANTs have also been shown to associate with heterologous partner proteins, including various subunits of the electron transport chain complexes, and form larger assemblies that range from 67 to 669 kDa (Lu et al., 2017; Chorev and Robinson, 2019). Additionally, ANTs have been observed to interact with several MCs, including P_i_T (Lu et al., 2017), and P_i_Ts have been proposed to function as both monomers and dimers (Schroers et al., 1998; Kunji and Crichton, 2010). Available evidence suggests that UCPs can exist in monomeric (Lee et al., 2015), dimeric (Lin et al., 1980) and tetrameric forms (Ardalan et al., 2021a; Hoang et al., 2013; Tabefam et al., 2024).

The densely packed protein environment of the MIM that results from its low lipid-to-protein ratio is likely to promote interactions between membrane proteins leading to the formation of homo- and/or hetero-oligomers (Schlame, 2021; Iovine et al., 2021). Indeed, an ability of MIM proteins to form higher-order complexes with one another may be a mechanism needed to deal with the stress caused by the high degree of protein crowding in the MIM (Schlame, 2021), as well as extramembranous environmental stress. While the exact functional significance of such oligomeric structures remains to be fully elucidated, it is plausible that they contribute to the regulation/optimization of protein activity within the membrane. For instance, association of a multitude of subunits results in the formation of respiratory supercomplexes involved in oxidative phosphorylation (Lapuente-Brun et al., 2013), optimizing substrate channeling and enhancing electron flow for efficient oxidative phosphorylation. Understanding the transient, flexible and dynamic nature of oligomerization and the possibility of interconversion between different oligomeric states (as well as dissociation and reassociation of oligomers), is therefore important for understanding the structure-function connections of MCs such as ANT, P_i_T and UCP.

The current study aimed to investigate the functional forms of ANT1, P_i_T, UCP2 and UCP4, both individually and in combination. Recombinant affinity-tagged versions of the proteins were expressed in *E. coli* (*Escherichia coli*) membranes, purified in their native-like forms as monomers, and reconstituted into mitochondrial membrane mimics with different lipid compositions. Protein conformation and self- and/or hetero-oligomerization were investigated and compared using circular dichroism (CD) spectroscopy and semi-native SDS-PAGE. Fluorescence spectroscopy-based proton transport assays were used to assess the function of reconstituted proteins. Proton transport rates of individual MC proteins were compared to rates when proteins were reconstituted in combinations, and comparisons were also made between different combinations of the proteins. Transport rates were also compared for proteins reconstituted in lipid membranes with or without cardiolipin, in the presence of fatty acid activators with different hydrocarbon chain lengths and degrees of saturation (i.e., oleic and palmitic acid) and in the absence and presence of inhibitors (i.e., ATP, GTP and CATR). Finally, comparisons were made for proton transport mediated by UCP4 and ANT1 that were reconstituted into liposomes together at different ratios. Collectively, the data suggest that collaborative interactions among this group of MC proteins (UCP2, UCP4, ANT1, PiT-B) differentially influence the proton transport activity across MIM and potentially play an important role in mitochondrial energetics.

## 2 Materials and methods

### 2.1 Chemicals

L-α-phosphatidylcholine (L-α-PC), extracted from dry egg yolk was purchased from Sigma-Aldrich (St. Louis, MO). This mixed lipid extract contains more than 60% (w/w) PC; the remaining 40% consists mostly of phosphatidylethanolamine (PE) and small amounts of other lipids. Cardiolipin [18:1; 1′,3′-bis [1,2-dioleoyl-snglycero-3-phospho]-glycerol (sodium salt)] was obtained from Avanti Polar Lipids (Alabaster, AL), octyl glucopyranoside (OG) was from BioShop Canada Inc. (Burlington, ON), and octyltetraoxyethylene (C_8_E_4_) was from Bachem (Bubendorf, Switzerland). The fluorescent probe dye, SPQ (6-methoxy-N-(3-sulfopropyl) quinolinum; 99% purity) was obtained from Biotium Inc. (Fremont, California). Carboxyatractyloside (potassium salt) was purchased from Cayman Chemical (Michigan, USA). All other chemicals were purchased from Sigma (St. Louis, MO) unless otherwise indicated.

### 2.2 Overexpression, membrane extraction and purification of proteins

cDNAs encoding proteins of interest were cloned into the pET26b (+) expression vector, such that recombinant versions of bovine ANT1 and human P_i_T-B, UCP2 and UCP4 (UniProt accession codes: ANT1 - P02722, P_i_T-B - Q00325-2, UCP2 - P55851, and UCP4 - O95847), were produced with a pelB leader sequence, followed by a His_6_-tag, fused at the N-terminus. Expression of recombinant fusion proteins was achieved in *E. coli* BL21 (DE3) or BL21 CodonPlus (DE3)-RIPL cells using a modified autoinduction method as previously described (Hoang et al., 2013; Tabefam et al., 2024; Studier, 2005). Briefly, the bacterial cells were grown overnight in 500 mL of autoinduction culture media (1% tryptone, 0.5% yeast extract, 1 mM MgSO_4_, 0.5% glycerol, 0.05% glucose, 0.2% lactose, 25 mM (NH_4_)_2_SO_4_, 50 mM KH_2_PO_4_, 50 mM Na_2_HPO_4_) at room temperature. After 22 h, the culture was centrifuged at 4000 *g* for 1 h at 4 °C using a JLA 10.500 rotor (Beckman Coulter) and the cell pellets were resuspended in lysis buffer [500 mM NaCl, 5 mM MgCl_2_, 20 mM Tris-HCl pH 8.0, one-quarter of a tablet of enediaminetetraacetic acid (EDTA)-free protease inhibitor (Roche Applied Science), one small scoop of DNase, and lysozyme (BioShop Canada Inc.) at a final concentration of 0.1 mg/mL]. A high-pressure cell disruptor (Constant Systems Limited, Daventry, U.K.), operating at 20 kPsi, was used to lyse the cells. The cell lysate was centrifuged at 20,000 × g for 20 min in a JA 25.5 rotor (Beckman Coulter) to remove intact cells, inclusion bodies or aggregated proteins. The cleared supernatant was then ultracentrifuged at 50,000 × g (MLA 80 rotor, Beckman Coulter) for 1 h to obtain the bacterial membranes in the pellet fraction.

Purification of proteins in their monomeric form from the bacterial membrane fraction was achieved using immobilized metal ion affinity chromatography (IMAC) via the procedure described in detail previously (Tabefam et al., 2024). Briefly, binding buffer containing 10 mM imidazole, 1% (w/v) lauryldimethylamine oxide (LDAO detergent), 500 mM NaCl, 1 mM Tris (hydroxypropyl) phosphine (THP), and 20 mM Tris-HCl, pH 8.0 was used to solubilize the bacterial membrane pellets containing the His-tagged recombinant protein and the suspension was incubated overnight at 4 °C on a rotating mixer. The solution was then incubated while mixing with 2 mL of nickel-nitrilotriacetic acid (Ni-NTA) resin (Bio-Rad, Waltham, Massachusetts) that had been equilibrated and suspended in binding buffer in a column for 1 h. The resin was allowed to settle, the flow-through was collected, and the resin was washed with 8 mL of binding buffer. Detergent exchange (from 1% LDAO to 1% OG) and further washing was accomplished by applying 5 mL of wash buffer containing 30 mM imidazole, 1% (w/v) OG, 500 mM NaCl, 1 mM THP, and 20 mM Tris-HCl, pH 8.0. Stepwise elution was achieved by applying elution buffer (1% (w/v) OG, 500 mM NaCl, 1 mM THP, and 20 mM Tris-HCl, pH 8.0) containing increasing concentrations of imidazole [100 mM (2 mL), 250 mM (2 mL), and 400 mM (6 mL, collected as six 1 mL fractions)]. Econo-Pac 10DG desalting spin columns (Bio-Rad, Hercules, California) were used to achieve removal by buffer exchange of 400 mM imidazole from the elution fractions containing the recombinant protein. For CD spectroscopic measurements the proteins were desalted into CD buffer (20 mM Tris-HCl, pH 8.0, 50 mM NaCl, 1% OG and 1% glycerol). For proton transport assays the desalting buffer was composed of 1.5X internal buffer [120 mM TEA_2_SO_4_, 45 mM TEATES (TEA: tetraethylammonium, TES: N- [tris(hydroxymethyl)methyl]-2-aminoethanesulfonic acid anion), 1.5 mM EDTA, pH 7.2] supplemented with 1% OG. The identity of the purified proteins was confirmed by Western blotting and MS analyses as described previously (Tabefam et al., 2024); furthermore, MS was used to confirm that the pelB leader sequence had been removed, presumably by the endogenous protein trafficking system in *E. coli* (Tabefam et al., 2024). Purity, and concentration of the purified proteins was assessed using semi-native sodium dodecyl sulfate-polyacrylamide gel electrophoresis (SDS-PAGE) and modified Lowry assay (Peterson, 1977), respectively. Freshly desalted proteins were used immediately for functional and structural analyses.

### 2.3 Reconstitution into lipid vesicles for structural and functional analysis

For CD spectroscopic and proton transport measurements, native-like monomeric forms of purified recombinant His-tagged ANT1, P_i_T, UCP2 and UCP4 were reconstituted into lipid vesicles to form proteoliposomes using a previously described detergent-mediated reconstitution procedure (Tabefam et al., 2024; Jabůrek and Garlid, 2003), with minor modifications. Liposomes with two different lipid compositions were used in this study. All liposomes contained L-α-PC from egg yolk extract (∼60% PC) (EYPC), either in the absence or presence of 18:1 cardiolipin (CL) added to a final concentration of 2.5 mol%. Briefly, a thin layer of lipid film, dissolved in methanol/chloroform (1:3) was formed in a round-bottomed flask under vacuum overnight. The lipid was then rehydrated in the desired reconstitution buffer to form multilamellar liposomes. For CD spectroscopic analysis, the reconstitution buffer contained 20 mM Tris-HCl, pH 8.0, 50 mM NaCl, 1% glycerol. For proton transport experiments, 1X internal buffer supplemented with the fluorescent SPQ probe at a final concentration of 3 mM was used at pH 7.2. The multilamellar liposomes were then solubilized in C_8_E_4_ to a final detergent/phospholipid ratio of 2.5 (w/w). Purified, desalted monomeric proteins were then added to the mixed lipid/detergent micelles. Unilamellar proteoliposomes were formed spontaneously following the removal of C_8_E_4_ detergent using SM-2 Biobeads (Bio-Rad). For proton transport assays, external SPQ probe was removed using a coarse Sephadex G25-300 (GE Healthcare) spin column. For CD spectroscopy studies, the final protein:phospholipid molar ratio was on the order of 1:1000 and in proton transport experiments this ratio was 1:10,000. In each experiment, protein-free liposomes were prepared in parallel as a control.

### 2.4 Semi-native SDS-PAGE

Semi-native SDS-PAGE was used to assess the oligomeric state of purified recombinant proteins before and after reconstitution into liposomes. In this technique, the amount of sodium dodecyl sulfate (SDS) is significantly reduced as compared to more traditional denaturing SDS-PAGE to provide the “semi-native” conditions (Voulhoux et al., 2003). Specifically, SDS is omitted from sample loading buffer, and is only included in the gel and running buffer at a concentration of 0.1% w/v (as compared to 1% w/v in fully denaturing conditions). In addition, protein samples were not heated and only incubated at room temperature for 5–10 min prior to loading on the gel. Gels were run at 110 V, stained with 0.2% (w/v) Coomassie Brilliant Blue R-250 in methanol: acetic acid: water (45:10:45 by volume) for 30–60 min and destained for 2 h.

### 2.5 Liposome size measurements

The size and homogeneity of liposomes and proteoliposomes were determined by dynamic light scattering (DLS) using a Zetasizer Nano ZS (Malvern Instruments, Worcestershire, U.K.). The average radii of prepared proteoliposomes for the proton transport assays and CD measurements were about 80 nm. On average, the radii of the blank liposomes were ∼10% larger than the proteoliposomes. The results were the average of 3-5 measurements.

### 2.6 CD spectroscopic measurements

Far-UV CD measurements were performed at 25 °C, at 1 nm resolution, on an AVIV 215 spectropolarimeter (Aviv Biomedical, Lakewood, New Jersey). Quartz cells with 0.1 cm path length were used for measuring the CD spectra of proteins in OG detergent or reconstituted into liposomes. Ellipticities were converted to mean residue ellipticity, [*θ*]. All individual CD spectra were an average of at least two independent preparations each measured 3 times, for a total of 6 measurements. The concentrations of proteins were 8–10 µM for samples in 1% OG and ∼1 µM in proteoliposomes.

### 2.7 Proton transport measurements

Proton efflux mediated by proteins reconstituted in lipid vesicles was measured by the anion-sensitive fluorescence quenching method, in which anions quench the fluorescent dye SPQ by a dynamic collision mechanism, as described previously (Jabůrek and Garlid, 2003; Jabůrek and Garlid, 2003; Hoang et al., 2012). The rate of proton efflux is measured indirectly through the quenching of SPQ by TES^−^. In a low pH environment, TES (pK_a_ ∼7.4) is fully protonated and does not affect the fluorescence of SPQ. However, when a proton is lost at high pH, the anionic TES (TES^−^) quenches SPQ fluorescence (Orosz and Garlid, 1993). These steady-state fluorescence measurements were performed in a Cary Eclipse spectrophotometer (Varian, Palo Alto, CA) with a bandwidth slit of 5 nm and a scan speed of 600 nm/min. Excitation and emission of the SPQ fluorescence signal were at 347 and 442 nm, respectively. The fluorescence scans were performed at 25 °C. Each proton transport analysis was an average of at least 5 independent measurements. For each proton transport assay, 40 µL of (proteo-) liposomes was added to 1.96 mL of external buffer [K_2_SO_4_ (80 mM), KTES (30 mM), EDTA (1 mM), pH 7.2]. The internal buffer of the liposomes consisted of TEA_2_SO_4_ (80 mM), TEATES (30 mM), and EDTA (1 mM), pH 7.2. The osmotic pressure was also kept equal across the membrane at the beginning of the measurements. Palmitic acid (as palmitate, PA) or oleic acid (as oleate, OA) were dissolved in 70% ethanol (with sonication, as necessary) and were added to a final concentration of 100 µM to activate proton efflux by the reconstituted carrier protein(s) in proteoliposomes (Hoang et al., 2012). The addition of the K^+^ ionophore valinomycin (val) facilitates the influx of K^+^, which in turn triggers fatty acid-dependent proton efflux by carrier proteins to offset the osmotic discrepancy in membrane potential. Proton efflux results in deprotonation of TES buffer (to form TES^−^), which quenches SPQ fluorescence. The rate of decrease in the SPQ fluorescence signal was correlated to the rate of fatty acid-activated proton transport by the protein under study. The latter was measured by monitoring the decrease in SPQ’s fluorescence signal intensity within the first 30s after the addition of valinomycin (Supplementary Figure S1) (Hoang et al., 2013). All proton transport data in liposomes were corrected for basal nonspecific leak by subtracting the proton efflux measured for blank (protein free) liposomes. Moreover, the proton transport data were calibrated for the SPQ fluorescence response, internal volume of proteoliposomes, and protein concentration (Jabůrek and Garlid, 2003). The concentration of protein in proteoliposomes was determined using a modified Lowry assay, as described previously (Peterson, 1977; Hoang et al., 2012). No significant proton transport/leakage was observed for the proteoliposomes in the absence of valinomycin. The influence of purine nucleotides ATP and GTP and the ANT-specific inhibitor CATR on proton transport was assessed by incubating proteoliposomes with 500 µM ATP, 500 µM GTP or 5 µM CATR for 2.5 min prior to addition of valinomycin.

### 2.8 Statistical analysis

All data analyses were performed using SAS version 9.4 (SAS Institute, 2012). Residuals were visually assessed to confirm homogeneity of variance, and normality was tested using the Shapiro-Wilk and Kolmogorov-Smirnov tests. Proton efflux rates were statistically compared among 1:1 binary protein combinations, inhibition treatments, stoichiometric ratios, and between fatty acid activators and cardiolipin presence or absence with general linear models (e.g., ANOVA, t-tests) and general linear mixed models. General linear mixed models were used when there were random effects, which included experiment replicate (all tests), and in some cases cardiolipin presence/absence, type of fatty acid activator, and inhibition treatment. Interactions between fixed factor variables were initially included, and non-significant terms (including interactions) were removed from the models using backward selection. Significant main (fixed) effects with more than two treatments were further analyzed using *post hoc* Tukey’s tests to evaluate pairwise differences.

## 3 Results

### 3.1 Expression and purification of UCP2, UCP4, ANT1 and P_i_T

**FIGURE 2:**
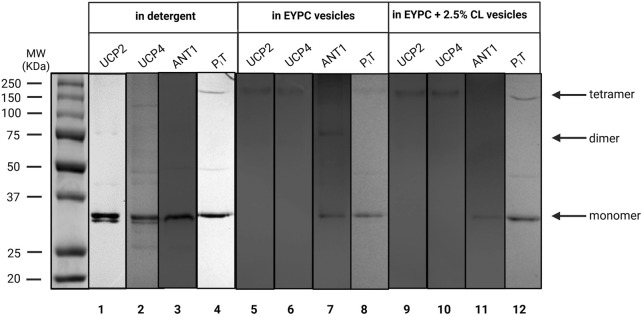
Semi-native SDS-PAGE analysis of purified recombinant UCP2, UCP4, ANT1, and P_i_T in OG detergent (lanes 1–4) and reconstituted in liposomes of different compositions; egg yolk extract (∼60% PC) (EYPC) (lanes 5–8), and EYPC +2.5% added CL (lanes 9–12) detected by Coomassie blue staining. The arrows indicate the positions of protein monomers, dimers, and tetramers. Vertical lines indicate lanes taken from separate gels. A representative molecular weight marker (kDa) lane is shown on the left.

Subcloning the cDNAs encoding the four proteins of interest (UCP2, UCP4, ANT1 and P_i_T) into the pET26b (+) expression vector that incorporates the pelB leader sequence followed by a six-histidine affinity tag (His_6_) in-frame with the N-termini of the proteins, combined with the application of a modified autoinduction expression method, resulted in targeting of the proteins to the inner membrane of *E. coli* and enabled purification of the proteins using immobilized metal affinity chromatography. The pelB leader sequence is a signal peptide that typically targets secreted proteins to the periplasmic space where it is cleaved by signal peptidase (Tabefam et al., 2024). However, the hydrophobic nature of transmembrane domains of the carrier proteins used here hinders complete translocation, resulting in insertion into the membrane due to their hydrophobic interactions with the bacterial membrane (Hoang et al., 2013). The membrane fractions were then isolated using differential velocity centrifugation. Mild detergents were used to extract the proteins from the membrane and resulted in copurification of proteins with closely associated membrane lipids. This lipid shield protects proteins from potential denaturing interactions with the solubilizing detergent and maintains the proteins in a relatively folded/native state (Hoang et al., 2013; Tabefam et al., 2024). The His_6_-tagged proteins were purified using nickel-containing affinity columns as monomers in the presence of OG detergent. OG was selected based on our previous study on conformational and thermal stability analysis of UCP1 in different detergents which revealed that the overall integrity of the protein was most stable when purified and stored in 1% OG micelles (Hoang et al., 2013). Purity of the proteins was confirmed by semi-native SDS-PAGE (Figure 2, lanes 1–4). The identity of each protein was confirmed by Western blotting, using monoclonal antibodies for UCP4 and P_i_T and a polyclonal antibody for ANT, and MS spectrometry as described previously (Tabefam et al., 2024).

### 3.2 Semi-native SDS-PAGE analysis of pure proteins and their combinations before and after reconstitution

As shown in Figure 2, all proteins existed in their monomeric forms when purified from *E. coli* membrane extracts in the presence of mild OG detergent. As shown previously, ANT, PiT and UCP4 migrate similarly on semi-native PAGE, despite their modest molecular weight differences, which can be detected when the proteins are compared using fully-denaturing SDS-PAGE (Tabefam et al., 2024). To investigate the behaviour of each protein when reconstituted into liposome membranes, purified proteins were initially reconstituted individually in liposomes made using an egg yolk lipid extract in either the absence or presence of added cardiolipin (EYPC ±2.5% CL) for structural and proton transport functional analyses. Egg yolk extract was chosen over other lipid systems as it contains a majority of PC (∼60%) along with a mixture of other phospholipids (such as PE) that are found in the MIM in similar proportions (Horvath and Daum, 2013). The effect of added CL was examined as it is a characteristic lipid found exclusively in the MIM (Daum, 1985). The semi-native electrophoretic analysis revealed interesting findings regarding the behavior of each protein when reconstituted into liposomes. The high molecular weight bands observed in Figure 2 (lanes 5, 6, 9, and 10) show that UCP2 and UCP4 tend to self-associate into tetramers when reconstituted into liposomes regardless of the presence or absence of added CL. Conversely, reconstituted P_i_T, in both the absence and presence of CL, remained mainly in its monomeric form, consistent with its state in the original OG detergent (Figure 2, lanes 8 and 12). The behavior of ANT1, on the other hand, varied depending on the lipid composition; when reconstituted in liposomes composed of EYPC in the absence of added CL, ANT1 remained predominantly in its monomeric state, with a weak indication of self-association into dimeric forms. However, when reconstituted in the presence of 2.5% CL, the protein appears to remain exclusively monomeric as the faint shadow of the dimeric form disappeared (Figure 2, compare lanes 7 and 11). It is assumed that all proteins had closely-associated native lipids when extracted and purified from *E. coli* membranes, which may have included an unknown amount of CL; therefore proteins reconstituted into liposomes without additional CL likely contained some residual CL, and those reconstituted in the presence of 2.5% CL may have had a final concentration of CL that was slightly higher than 2.5%.

**FIGURE 3:**
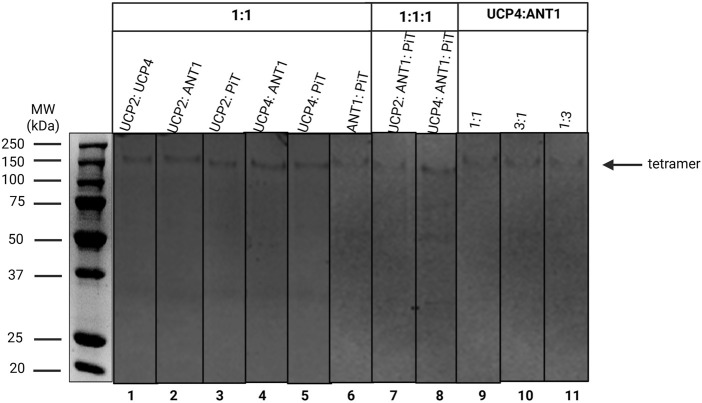
Semi-native SDS-PAGE analysis of binary (lanes 1–6) and ternary (lanes 7 and 8) stoichiometric combinations of carrier proteins reconstituted in liposomes detected by Coomassie blue staining. Lanes 9-11 correspond to the 1:1, 3:1 and 1:3 stoichiometric ratios of the UCP4: ANT1 pair. The arrow indicates the location of protein tetramers. Vertical lines indicate lanes taken from separate gels. A representative molecular weight marker (kDa) lane is shown on the left.

The current study focused on assessing whether binary and ternary combinations of these proteins, in various stoichiometric ratios, would associate and form hetero-oligomers upon reconstitution in lipid vesicles. As illustrated in Figure 3 (lanes 1–6), regardless of lipid composition, all 1:1 stoichiometric binary combinations of carriers that were tested (UCP2:UCP4, UCP2:ANT1, UCP2:P_i_T, UCP4:ANT1, UCP4:P_i_T, and ANT1:P_i_T) formed tetramers. Interestingly, binary combinations including ANT1 and P_i_T, which form mainly monomers when reconstituted on their own (Figure 2, lanes 7-8 and 11-12, respectively), whether reconstituted with each other or with UCP2 or UCP4, also led to the formation of tetramers (Figure 3, lanes 2–6). That ANT1 and P_i_T do not form homo-tetramers, and that tetramers are only observed when these proteins are co-reconstituted in binary combinations with other carrier proteins, indicates that the tetramers that are formed from binary combinations include both proteins (i.e., they are heterotetramers). Reconstitution of three proteins together in 1:1:1 stoichiometric combinations of proteins (UCP2:ANT1:P_i_T and UCP4:ANT1:P_i_T) also resulted in the formation of tetramers (Figure 3, lanes 7 and 8).

### 3.3 CD spectroscopic conformational analyses of UCP4, ANT1 and P_i_T

**FIGURE 4:**
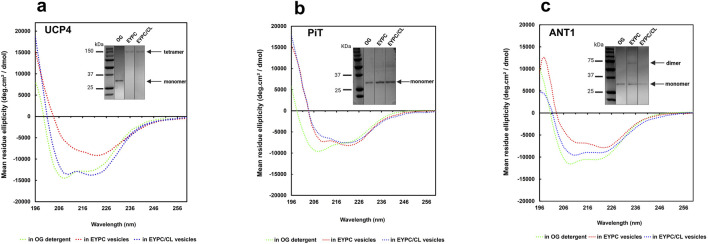
CD spectra of purified recombinant **(a)** UCP4, **(b)** P_i_T, and **(c)** ANT1 in OG detergent and reconstituted in liposomes of varying lipid composition (EYPC ±2.5% added CL) at 25 °C. Protein concentrations were 8–10 μM in OG and **∼** 1 μM in lipid vesicles. Insets (taken from Figure 2, shown again here for reference) show the semi-native PAGE analysis of UCP4, P_i_T, and ANT1 in OG and in lipid systems with (EYPC/CL) and without added CL (EYPC). Vertical lines indicate lanes taken from separate gels. Molecular weight markers (kDa) are shown in lanes on the left.

Circular dichroism (CD) spectroscopy is a sensitive technique for studying protein conformations and monitoring structural changes in proteins. In this study, CD was utilized to characterize and compare the conformations of UCP4, ANT1 and P_i_T proteins in OG and liposomes of different lipid compositions (EYPC ±2.5% CL) (Figure 4). CD spectra of the proteins in the far UV region (below 250 nm), dominated by the electronic transitions of the amide (peptide) bond chromophore, give information about protein secondary structures. Irrespective of the environment (detergent/lipid membrane), the CD spectra of all proteins exhibited distinctive spectral features of α-helical backbone structures [negative maxima at 208 nm and 222 nm, and a positive maximum around 193 nm (not shown), corresponding to π → π* (193 and 208 nm) and n → π* (222 nm) transitions of the peptide bond] (Greenfield, 2006). It is worth mentioning that light scattering and flattening effects (Hoang et al., 2012), that limit the CD studies of membrane proteins, did not distort the spectra in the wavelength range shown in Figure 4.

UCP4 tetramers in EYPC liposomes (Figure 4a, inset) displayed a notable reduction in the far-UV CD signal intensity compared to the monomeric protein in OG detergent, indicating a reduced degree of helicity (Figure 4a). However, inclusion of 2.5% CL in the lipid membrane led to an enhancement in helicity and in the tetramers comparable to the helicity observed for the monomers in OG, albeit with differences in the CD spectra shape (Figure 4a). These results suggest a significant conformational change of the protein from OG (monomers) to EYPC vesicles (more densely-packed monomeric units in tetramers) and subsequently from EYPC to EYPC +2.5% CL vesicles (more relaxed or less densely packed monomeric units in tetramers), as evidenced by a notable decrease in negative ellipticity and a reversal of the relative intensity of minima (θ_208_/θ_222_) from 1.18 (in OG) to 0.98 (in EYPC +2.5% CL liposomes) and 0.6 (in EYPC liposomes). These findings underline the importance of the lipid environment in the folding and structural stabilization of UCP4.

The differences between tetrameric UCP4 in liposomes and its monomeric form in OG are further evident from the shape of the CD spectra of the two preparations. In particular, when reconstituted in EYPC liposomes, the CD spectrum of UCP4 tetramers displayed a distinct shoulder-like π → π* parallel transition band around 208 nm, replacing the very intense minimum observed for monomers in OG. Additionally, a more intense n → π* transition band emerged at 222 nm (Figure 4a). This shift is reflected in the θ_208_/θ_222_ ellipticity ratio, which transitioned from 1.18 for the UCP4 monomer in OG to 0.60 upon reconstitution in EYPC liposomes (Figure 4a). A ellipticity ratio below 1.0 is consistent with previous findings regarding the packing density in helical bundle motifs and self-associated oligomers of human UCPs (Hoang et al., 2015). The CD spectra for UCP4 tetramers in this study align well with spectra reported in previous studies (Hoang et al., 2015).

Compared with UCP4, the transition of the θ_208_/θ_222_ ellipticity ratio for P_i_T from 1.27 in the original state (OG) to 0.87 after reconstitution in EYPC vesicles, and further decrease to 0.77 in the presence of 2.5% CL, together with apparent changes in the shape of the CD spectra, indicate a conformational shift of the monomeric form when reconstituted into the lipid environment (Figure 4b). These alterations suggest a potential change in the compactness of the helices within each monomeric unit in the lipid environment.

For ANT1, the θ_208_/θ_222_ ellipticity ratio changed from 1.11 in OG detergent to 0.81 when reconstituted in EYPC vesicles, and 1.07 in the EYPC +2.5% CL lipid environment. These observations correlate with the semi-native gel results (Figure 4c, inset), which indicate that reconstitution in EYPC vesicles led to some degree of oligomerization (evidenced by a faint dimeric band). However, ANT1 remained monomeric when reconstituted in liposomes that included 2.5% CL in the lipid environment similar to what was observed in OG, but with a reduced helical intensity (Figure 4c). The reduced helical intensity of the reconstituted protein is indicative of denser packing of helices in monomeric units.

A comprehensive study on UCP2, which included CD analysis, was recently published by our group, and suggested that the increased ellipticity observed for tetrameric UCP2 compared to UCP2 in OG was likely due to a higher degree of helicity after reconstitution in liposomes (Ardalan et al., 2021a; Ardalan et al., 2021b). In summary, based on our experimental conditions the differences between the CD spectra of UCP4, P_i_T and ANT1 show that these proteins acquire different molecular forms and different degrees of helical packing after reconstitution in vesicles of different lipid compositions. For example, in EYPC liposomes, the lower θ_208_/θ_222_ ratio of UCP4 compared to ANT1 and P_i_T (0.60 vs. 0.81 and 0.87) is consistent with the semi-native SDS-PAGE data suggesting that UCP4 forms tetramers when reconstituted into liposomes, whereas ANT1 and P_i_T remain mainly monomeric.

### 3.4 Proton transport of carrier proteins reconstituted alone and in combination

To study the potential formation and function of heteromeric carrier protein complexes, the rate of proton efflux was compared for individually reconstituted proteins and combinations of the carrier proteins that were reconstituted at different stoichiometric ratios. Proton efflux was measured using a fluorescence quenching based assay, as described previously (Hoang et al., 2012). The proton efflux rate (PER) is expressed as µmol proton efflux/min/mg protein (for instance, PER = 16.3 ± 0.3) (Supplementary Figure S1) for proteins reconstituted in liposomes.

In several experiments, proton efflux was quantified and compared between experimental systems with and without added cardiolipin [EYPC ±2.5% CL]. Data were also collected and compared in the absence or presence of two different free fatty acids (FAs), palmitic acid (as sodium palmitate, PA) or oleic acid (as sodium oleate, OA). PER for all proteins reconstituted into liposomes in the absence of added FAs was comparable to blank liposomes (data not shown). The inhibitory effects of purine nucleotides (ATP and GTP) and the ANT c-state-specific substrate inhibitor, CATR, on selected proteins and/or selected combinations of proteins were also examined.

#### 3.4.1 Effects of fatty acids, cardiolipin, and nucleotides on individual carrier protein transport

The presence of cardiolipin had an overall significant and positive effect on proton transport, when variation due to protein identity, type of FA activator (OA or PA) and inhibition treatment was accounted for (General linear mixed model (GLMM), F_1,88_ = 41.32, P < 0.0001). Based on our previously reported data on the influence of FAs of differing geometry and structure on the proton transport of neuronal UCPs (Hoang et al., 2015), saturated PA (16:0) and unsaturated OA (18:1 Δ^9^) were used as activators in the current study, and proton transport was compared in the presence of these two FAs. Data showed that all pure proteins and their combinations were able to transport protons only after the addition of the FAs, consistent with earlier findings regarding the inability of UCP (Hoang et al., 2012; Orosz and Garlid, 1993; Horvath and Daum, 2013), ANT (Brand et al., 2005; AYu et al., 1989; Kreiter et al., 2021) and P_i_T (Engstová et al., 2001) (in proteoliposomes and mitoplasts) to conduct proton transport in the absence of FAs.

**FIGURE 5:**
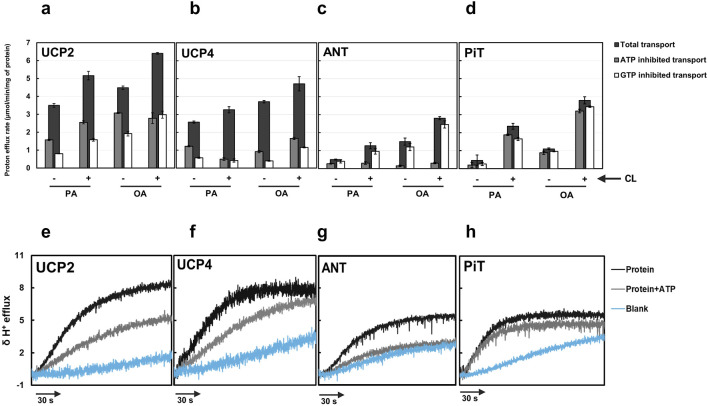
Total proton transport rates in the absence and presence of ATP and GTP for individual proteins [**(a)** UCP2, **(b)** UCP4, **(c)** ANT1, and **(d)** P_i_T)] in liposomes [EYPC with (+) and without (−) 2.5% added CL] and in the presence of palmitic acid (PA) or oleic acid (OA). Each experiment included final concentrations of 1 μM protein, 1 mM lipid, 100 μM fatty acid (PA or OA), and in cases where nucleotides were added, 500 μM ATP or GTP. Dark bars represent the total proton transport rate in the absence of purine nucleotides, Gray bars represent proton transport rate in the presence of ATP, and white bars represent the proton transport rate in the presence of GTP. Each bar represents the average transport rate of at least 10 repeats (from at least two independent experiments), and the error bars show the standard deviations. For numerical values, see Supplementary Tables S1, S2. The statistical analysis of the data presented in this figure is addressed in the text. Representative traces showing the proton efflux rates of individual proteins reconstituted in the presence of added 2.5% CL and with PA as the fatty acid in the absence of nucleotide and presence of ATP for each individual protein are shown in panels **(e–h)**. The rate of proton leak measured for protein-free liposomes is also shown in each graph.

Results indicated that both OA and PA interacted with all proteins and their combinations, and influenced proton transport activities differently. For each individual protein, OA was a significantly more potent activator than PA (Figures 5a–d; Supplementary Tables S1, S2, S4, S5) (General linear models (GLMs), F_1,13_ > 48.38, P < 0.0001). The total proton transport rates for UCP2 and UCP4 in the presence of PA were PER = 3.5 ± 0.1 and PER = 2.6 ± 0.06, respectively. These rates increased to PER = 4.50 ± 0.10 and PER = 3.70 ± 0.07 in the presence of OA (Figures 5a,b; Supplementary Table S1). In contrast, with PA as the proton transport activator, both ANT1 and P_i_T exhibited minimal (nearly zero) basal leak, with transport rates of PER = 0.50 ± 0.03 and PER = 0.46 ± 0.30, respectively. However, when OA was added, their transport rates rose to PER = 1.50 ± 0.20 and PER = 1.10 ± 0.05, respectively (Figures 5c,d; Supplementary Table S1), underscoring the importance of FA type on activating the transport activity of individual proteins (Kreiter et al., 2021; Beck et al., 2007). This observation is consistent with our previously reported data on the FA dependency of proton transport function of neuronal UCPs (Hoang et al., 2015).

When effects of FAs, presence/absence of added CL, and presence/absence of inhibitors were accounted for, proton transport differed significantly among the four proteins (GLMM, F_2,85_ = 30.98, P < 0.0001). UCP2 had significantly higher proton transport than the other singular proteins (Tukey’s test, P < 0.05). The inclusion of 2.5% CL in the lipid system significantly increased proton transport activity of all tested singular proteins (GLMM, F_1,13_ > 17.04, P ≤ 0.0012) (Figures 5a–d; Supplementary Table S2, S5). The higher proton conductance in the presence of CL could be explained by the more relaxed packing of the monomers either as monomers or tetramers (Figure 4).

The inhibitory effect of purine nucleotides on proton transport was assessed for single proteins reconstituted in liposomes with and without added CL. The findings were consistent with previous reports, demonstrating that GTP is overall a more effective inhibitor than ATP of UCP2 and UCP4 (Figures 5a,b; Supplementary Tables S1, S2) (Krauss et al., 2005; Hoang et al., 2012; Woyda-Ploszczyca and Jarmuszkiewicz, 2014). For both UCP2 and UCP4, proton transport was significantly lower in GTP-inhibited systems than in either ATP-inhibited systems or the control (GLMM, F_2,13_ = 200.96, P < 0.0001). Inhibitory effects were modified in some cases, however, by presence of CL and identity of the FA activator.

For UCP2, the effect of inhibition was statistically consistent regardless of which FA activator (OA vs. PA) was present (i.e., no significant inhibition by (*) FA interaction; GLMM, F_2,13_ = 0.81, P = 0.46). There was, however, a significant cardiolipin*inhibition interaction (GLMM, F_2,13_ = 9.65, P = 0.0018). Investigation of this interaction (activators considered together) indicated that while proton efflux was significantly lower in the GTP-inhibited treatment than in the ATP-inhibited treatment when cardiolipin was absent (Tukey’s test, P < 0.05), there was no statistically significant difference between GTP- and ATP-inhibited treatments when cardiolipin was present (Tukey’s test, P > 0.05).

For UCP4, inhibition was affected by both identity of the FA activator (i.e., significant FA*inhibition interaction, GLMM, F_2,13_ = 10.55, P = 0.0019) and presence of added CL (i.e., significant cardiolipin*inhibition interaction, GLMM, F_2,13_ = 6.96, P = 0.0097). Whereas GTP was overall a better inhibitor than ATP, the difference in proton efflux by UCP4 in the presence of the two purine inhibitors was only significant when CL was present (Tukey’s test, P < 0.05). The difference in inhibition between GTP and ATP was also stronger in the presence of OA than in the presence of PA. These results underscore the likelihood of specific conformational changes in the carrier proteins induced by CL and particular FAs, which impact the ability of purine nucleotides to inhibit proton transport.

Regardless of the absence or presence of 2.5% CL in the liposome membranes, ANT1-mediated proton transport was significantly affected by the presence of inhibitors (GLMM, F_2,13_ = 160.57, P < 0.0001), and proton transport was inhibited by ATP more effectively than by GTP (Tukey’s test, P < 0.05) (Figure 5; Supplementary Table S1, S2). While ATP was a significantly better inhibitor than GTP with either OA or PA as a fatty acid activator (Tukey’s tests, P < 0.05), the difference between ATP- and GTP-inhibited systems was greater with OA than with PA; these results are consistent with previously reported data indicating that FA-activated proton leak by ANT is more effectively inhibited by adenine nucleotides compared to guanosine nucleotides (Kreiter et al., 2021).

While the overall proton transport activity of P_i_T was lower than for the other three single proteins, GTP and ATP inhibited P_i_T-mediated proton transport, and the effects depended on the presence of added CL. Transport was overall significantly affected by both inhibitors (P = 0.0003), and there was no significant difference in proton transport between GTP- and ATP-inhibited systems (P > 0.05). When CL was added, ATP and GTP affected significant and similar inhibition in P_i_T-mediated proton transport (P < 0.05). In the absence of added CL, however, neither ATP nor GTP had significant inhibitory effects on proton transport (P > 0.05). For P_i_T, effects of inhibitors did not vary between FA activators (i.e., no inhibition*activator interaction, p = 0.74).

Figures 5e–h show representative proton efflux rate traces for each individual protein reconstituted into liposomes in the presence of added CL (2.5%) and PA, and in the absence or presence of ATP. The rate of proton leak from protein-free liposome blanks are shown in each graph as well.

#### 3.4.2 Proton transport of binary protein combinations and ATP inhibition

**FIGURE 6:**
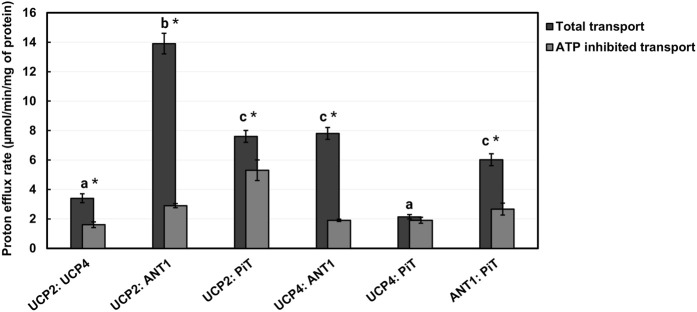
Proton transport rates of 1:1 stoichiometric combinations of proteins (UCP2:UCP4, UCP2:ANT1, UCP2:P_i_T, UCP4:ANT1, UCP4:P_i_T, ANT1:P_i_T) reconstituted in liposomes in the absence and presence of 500 μM ATP. Liposomes were composed of EYPC without added CL and transport was measured in the presence of 100 μM palmitic acid (PA). Each experiment included final concentrations of 1 μM total combined protein and 1 mM lipid. Dark bars represent the total proton transport rate in the absence of ATP, and Gray bars represent the proton transport rate in the presence of ATP (for corresponding numerical values, see Supplementary Table S3). Each bar represents the average transport rate of at least 10 repeats (from at least two independent experiments), and the error bars indicate standard deviations. The letters above the bars (a–c) indicate significant pairwise differences in total proton transport rate among the six protein combinations [Tukey’s tests (p ≤ 0.05)]. Significant differences between total and ATP-inhibited proton transport rate for each protein combination are indicated by asterisks.

After comparing the proton transport activities of the individual carrier proteins, experiments were conducted to test the hypothesis that different combinations of the proteins can form hetero-oligomers, and that proton transport activities are affected by heterotypic interactions. To assess protein-protein interactions between the various carrier proteins under study, we first reconstituted 1:1 binary combinations of the proteins in lipid vesicles of EYPC and measured the proton transport activity of the combinations in the presence of PA. The total proton efflux rates varied significantly across the six binary combinations tested (GLM, F_5,6_ = 185.24, P < 0.0001), with the UCP2:ANT1 pair showing the highest level of proton transport. Post-hoc analysis using Tukey’s tests confirmed that the proton efflux of UCP2:ANT1 was significantly greater than that of all other combinations (Tukey’s test, P < 0.05; Figure 6). In contrast, the UCP4:P_i_T combination exhibited the lowest proton efflux rate, and was significantly lower than all other combinations except UCP2:UCP4 (Tukey’s test, P < 0.05; Figure 6). The combinations UCP2:P_i_T, UCP4:ANT1, and ANT1:P_i_T showed no statistically significant differences in proton efflux (Tukey’s test, P > 0.05).

As shown in Figure 6; Supplementary Table S3, the binary combinations of ANT1 (GLMM, F_1,3_ = 804.6, P < 0.0001) or P_i_T (GLMM, F_1,3_ = 186.24, P = 0.0009) with UCP2 significantly increased proton transport, as compared to UCP2 alone; the addition of ANT1 or P_i_T increased the proton transport activity of UCP2 approximately fourfold and twofold, respectively. The existence of a respiratory-dependent interplay between ANT2 and UCP2 has been reported recently (Schiffer et al., 2022); although an interaction of UCP2 with ANT1 was not explored, an interaction of ANT1 with UCP2 is not surprising given the high degree of sequence identity between ANT1 and ANT2 (Pebay-Peyroula et al., 2003).

Whereas the proton transport rate for the combination of UCP2 and UCP4 (PER = 3.4 ± 0.3) was not significantly different than that for UCP2 alone (GLMM, F_1,3_ = 0.02, P = 0.8929) (compare Figures 5, 6), reconstituting UCP4 in a 1:1 ratio with ANT1 had a statistically significant positive effect on UCP4 proton transport rate (GLMM, F_1,3_ = 407.95, P = 0.0003), increasing it ∼3-fold (Figure 6). P_i_T, however, did not have a significant effect on proton transport of UCP4 (PER = 2.10 ± 0.10 for the combination as compared to PER = 2.60 ± 0.06 for UCP4 on its own) (GLMM, F_1,3_ = 4.96, P = 0.11) (Figures 5, 6; Supplementary Tables S2, S3). A surprising result was observed for the 1:1 combination of ANT1 and P_i_T; although both proteins exhibited a rate of proton transport that was comparable to minor basal leak on their own, the combination of the two proteins exhibited significantly higher proton transport activity (PER = 6.0 ± 0.4) compared to either ANT1 (GLMM, F_1,3_ = 351.31, pP = 0.0003) or P_i_T (GLMM, F_1,3_ = 268.36, pP = 0.0005) alone (Figure 6; Supplementary Table S3).

To further validate the heteromeric protein interactions and their implication for proton transport function, we also assessed the inhibitory effect of ATP on each 1:1 binary protein combination. Given that ATP inhibited the proton transport activity of UCPs and ANT1 but not of P_i_T, it was expected that reconstitution of P_i_T in combination with either UCP2 or UCP4 would result in complexes that were less susceptible to inhibition by ATP. Consistent with the inhibition observed for the individual proteins (Figures 5a–d; Supplementary Table S1), ATP significantly inhibited combinations of UCP2 or UCP4 with ANT1 by approximately 75% and 79%, respectively (t-tests, t > 24.5, P < 0.0017, df = 2) (Figure 6; Supplementary Table S3). In contrast, proton transport by each of UCP2 and UCP4 in combination with P_i_T was inhibited by ATP only by about 10%–30% (Figure 6; Supplementary Table S3) and while proton transport by UCP2 in combination with PiT was significantly inhibited by ATP (t-test, t = 16.64, P = 0.0036, df = 2), proton transport by UCP4 in combination with PiT was not significantly inhibited by ATP (t-test, t = 0.53, P = 0.65, df = 2). This finding generally aligns with the role of ATP as an effective inhibitor of the proton transport function of singular UCPs and ANT1, but not of P_i_T (Figures 5A–D; Supplementary Table S3). Moreover, the inhibitory effect of ATP on the combination of UCP4 and UCP2 was found to be significant (t-test, t = 7.27, P = 0.0018, df = 2) and similar to its effect on the proton transport function of each individual protein (∼53%) (Figures 5, 6; Supplementary Tables S1, S3). Similarly, for the combination of ANT1 and P_i_T, the significant inhibitory effect of ATP on the combination (t-test, t = 8.00, P = 0.0015, df = 2) closely resembled the inhibition observed when ATP was added to ANT1 reconstituted on its own (56%) (compare Figures 5, 6; Supplementary Tables S1, S3).

#### 3.4.3 Effects of stoichiometry on proton transport of UCP4: ANT1 heterotetramers

Building on our recent biphasic two-state model for proton transport by tetrameric UCP2 (a dimer of stable dimers), which proposes that all individual monomers are functional and monomers within each stable dimeric unit operate in the same transport mode (Ardalan et al., 2021b), we aimed to deepen our understanding of monomer associations in heterotetrameric carrier protein complexes and the contribution of each protein to proton transport in these combinations. Focusing on the UCP4:ANT1 pair, we reconstituted binary combinations of the two carrier proteins in different stoichiometric ratios to examine organization of monomers within the oligomer and each monomer’s contribution to proton transport. We selected three stoichiometric ratios (1:1, 3:1, and 1:3) and the UCP4:ANT1 pairs were reconstituted with these ratios in lipid systems composed of EYPC with and without 2.5% CL. Proton transport for each stoichiometric ratio was measured in the presence of PA and OA, and the inhibitory effects of ATP and GTP were determined. For the heterotetramers that were formed (Figure 3, lanes 9–11), we originally hypothesized that inclusion of a higher proportion of UCP4 would result in higher proton transport activity; predicting that the 3:1 combination would have the highest transport rate.

**FIGURE 7:**
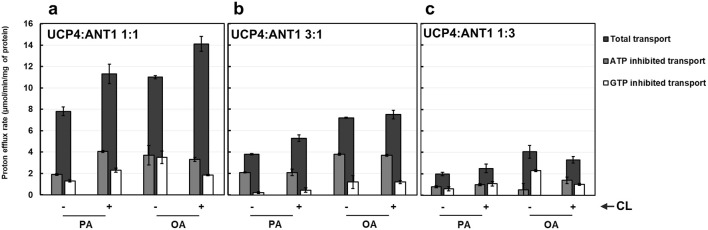
Proton transport of UCP4 and ANT1 co-reconstituted into liposomes at 1:1 **(a)**, 3:1 **(b)** and 1:3 **(c)** (UCP4:ANT1) stoichiometric ratios in the absence and presence of 500 μM ATP or GTP. Liposomes were made using EYPC with (+) or without (−) 2.5% added CL, and transport was measured in the presence of 100 μM palmitic acid (PA) or oleic acid (OA). Each experiment included final concentrations of 1 μM total combined protein and 1 mM lipid. Black bars represent the total proton transport rate in the absence of purine nucleotides, Gray bars represent proton transport rate in the presence of ATP, and white bars represent proton transport rate in the presence of GTP (for corresponding numerical values, see Supplementary Tables S4, S5). Each bar represents the average transport rate of at least 10 repeats (from at least two independent experiments), and the error bars show the standard deviations. The statistical analysis of the data presented in this figure is addressed in the text. Note that the data for the 1:1 UCP4:ANT1 ratio (PA, (−) CL, ± ATP) are reproduced from Figure 6.

Proton efflux differed significantly among UCP4 homotetramers and the three stoichiometrically different heterotetramers of UCP4:ANT1 (GLMM, F_3,87_ = 33.27, P < 0.0001). Contrary to expectation, the 1:1 ratio had the highest transport rates (e.g., 14.10 ± 0.70 in the presence of CL and OA); proton transport for the 1:1 stoichiometric ratio was significantly higher than for the 3:1 (e.g., PER = 7.50 ± 0.40) or 1:3 (e.g., PER = 3.30 ± 0.30) stoichiometric ratios, or for UCP4 on its own (PER = 4.5 ± 0.10) under the same conditions (Tukey’s tests, P < 0.05). The rates of transport by the 3:1 ratio of reconstituted UCP4:ANT1 was significantly higher (Tukey’s test, P < 0.05) than that of either the 1:3 ratio or UCP4-only, which did not significantly differ from each other (Tukey’s test, P > 0.05) (Figure 7; Supplementary Table S4, S5). The fact that there was no significant difference in proton transport between the 1:3 stoichiometric ratio and UCP4 alone (Tukey’s test, p < 0.05) indicates the importance of considering effects of stoichiometry on increasing proton transport in heterotetramers. Transport by UCP4-ANT1 heterotetramers varied from transport rates by ANT1 and UCP4 proteins alone. Transport by heterotetramers was also compared in the absence or presence of purine nucleotide inhibitors. The effects of GTP and ATP inhibition differed significantly among the 3 heterotetramers, UCP4, and ANT1 (GLMM, F_8,102_ = 42.05, P < 0.0001). Pairwise comparisons between heterotetramers and UCP4 revealed that in presence of the ATP inhibitor, both the 1:1 and 3:1 heterotetramers had significantly higher proton transport rates than either UCP4 or ANT1 alone (Tukey’s test, P < 0.05). In contrast, the 1:3 heterotetramer had similar proton transport to UCP4 and ANT1 when inhibited with ATP (Tukey’s test, P > 0.05). When GTP was present as an inhibitor, proton transport was similar between the 1:1 heterotetramer and ANT1 alone (Tukey’s test, P > 0.05), but significantly higher for the 1:1 heterotetramer than for UCP4 alone (Tukey’s test, P < 0.05). There was similar proton transport between the 1:3 heterotetramer and each of ANT1 and UCP4 in the presence of GTP (Tukey’s test, P > 0.05). Whereas the 3:1 heterotetramer had similar proton transport as ANT1 when GTP was present (Tukey’s test, P > 0.05), this heterotetramer had significantly lower proton transport than UCP4 when GTP was present (Tukey’s test, P < 0.05). Altogether, the proton transport results of UCP4-ANT1 heterotetramers prepared using different stoichiometric ratios of the proteins provides strong evidence for functional cooperativity between ANT1 and UCP4.

#### 3.4.4 Effects of CATR on ANT1: UCP heterotetramer proton transport

**FIGURE 8:**
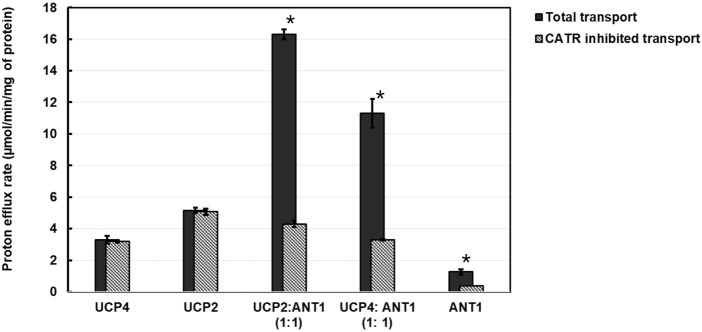
Proton transport and CATR-inhibited proton transport rates of reconstituted UCP2, UCP4, ANT1, co-reconstituted UCP4:ANT1 and UCP2:ANT1 (1:1 stoichiometric ratio) in liposomes made from EYPC containing 2.5% added CL, in the presence of 100 μM palmitic acid (PA). Each experiment included final concentrations of 1 μM total combined protein and 1 mM lipid. Dark bars represent the total proton transport rate in the absence of CATR, shaded Gray bars represent the proton transport rate in the presence of 5 μM CATR (for corresponding numerical values, see Supplementary Table S6). Each bar represents the average transport rate of at least 10 repeats (from at least two independent experiments), and the error bars show the standard deviations. Asterisks (*) indicate a statistically significant difference (p < 0.05) between the total proton efflux and CATR-inhibited efflux within each combination. Note that the proton transport data for UCP4, UCP2, and ANT1 are reproduced from Figure 5.

For the 1:1 combination of UCP4 and ANT1 heterotetramers, to further confirm the cooperativity between ANT1 and UCP4 dimers, we examined proton transport in the presence of the known ANT inhibitor CATR. CATR is an irreversible substrate inhibitor of ANT that blocks its ADP-ATP exchange function (Klingenberg et al., 1975). We surmised that irreversible inhibition of the nucleotide exchange activity of ANT and prevention of its alternating transport function could also affect the cooperativity of proton transport activity of ANT1-UCP4 heterotetramers. To verify this hypothesis, we first examined the inhibitory effect of CATR on the proton transport activity of individually reconstituted UCP4 and ANT1. In the lipid system that included 2.5% CL and in the presence of PA as a fatty acid activator, CATR did not significantly inhibit the proton transport activity of UCP4 homotetramers (t-test, t = 1.59, P = 0.25, df = 2) (Figure 8; Supplementary Table S6). However, CATR did significantly inhibit the proton transport observed for reconstituted ANT1 by approximately 70% (t-test, t = 6.62, P < 0.05, df = 2) (Figure 8; Supplementary Table S6). A similar inhibitory effect of CATR on the proton transport function of ANT has been reported in a previous study (Kreiter et al., 2021). More interestingly, in the UCP4:ANT1 (1:1) heterotetramer system, CATR significantly diminished (t-test, t = 16.26, P = 0.0038, df = 2) the proton transport rate from PER = 11.30 ± 0.90 to PER = 3.20 ± 0.20 (approx. 71% reduction), which is comparable to the proton transport rate of UCP4 on its own (PER = 3.29 ± 0.17) (Figure 8; Supplementary Tables S7, S8).

To extend the examination into the cooperative interaction observed between ANT1 and UCP4 to other UCP proteins, we examined the effect of CATR on a 1:1 combination of UCP2 and ANT1 under the same experimental conditions (presence of 2.5% CL and PA). The results indicated that, similar to UCP4, proton transport by UCP2 alone was not significantly inhibited by CATR (t-test, t = −0.94, P = 0.45 df = 2) (Figure 8; Supplementary Tables S7, S8). As was the case for the UCP4:ANT1 system, the proton transport rate of the UCP2:ANT1 heterotetramer was significantly reduced by CATR (t-tests, t ≥ 6.26, P ≤ 0.022, df = 2) from PER = 16.30 ± 0.30 to PER = 4.30 ± 0.20 (approximately 74% inhibition) (Figure 8; Supplementary Table S6). Collectively, these data not only support the existence of functional heterotetramers comprised of ANT1 and UCPs but also highlight the possibility of functional cooperativity between different UCP isoforms and ANT1. The significant reduction in proton transport in the presence of CATR suggests that the function of the individual proteins within UCP-ANT1 heterotetramers are maintained and can be modulated by the other member(s) of the complex.

## 4 Discussion

Although MC protein family members are essential for transporting a diverse array of substrates across the MIM, their functional molecular structures are still debated. Furthermore, despite their critical function in mediating the transport of various interrelated metabolites, the potential interactions between different MC proteins are largely understudied, leaving a gap in our understanding of their potential cooperative dynamics. This is mainly due to methodological limitations in studying live cells and/or challenges associated with isolating and purifying adequate quantities of stable protein complexes from mitochondria. In the current study, we focused on MCs involved in cellular energy regulation and processing: ANT1, P_i_T-B, UCP2 and UCP4. The study had two main objectives: the first was to determine the functional form(s) of individual recombinant proteins reconstituted into liposomes, and the second was to investigate the possibility of interactions among carrier proteins that form molecular complexes as hetero-oligomers and affect proton transport.

### 4.1 Molecular forms of proteins in proton transport

**FIGURE 9:**
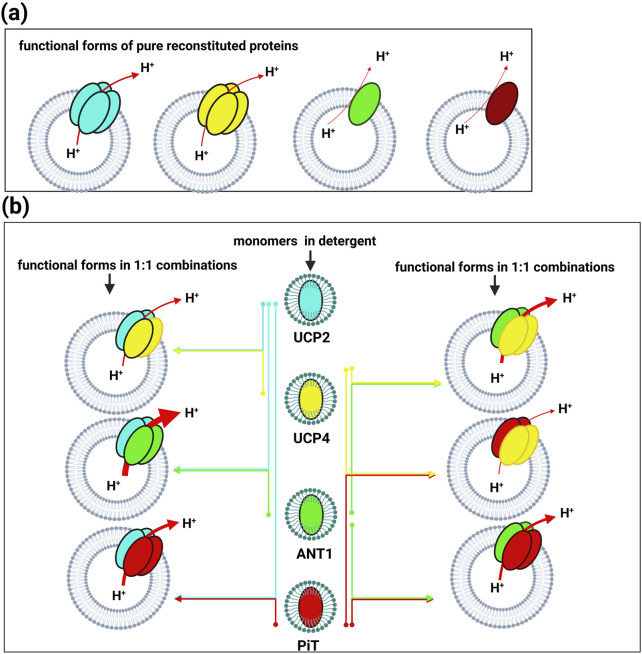
**(a)** Formation of functional heterotetrameric complexes (dimers of two different homodimers) when purified monomeric proteins (in detergent) are reconstituted in lipid vesicles in a 1:1 stoichiometric combination. **(b)** Pure proteins in lipid vesicles adopt distinct functional forms. Both UCP2 and UCP4 self-associate as tetramers, while both ANT and P_i_T remain in their monomeric forms. The red arrows indicate proton efflux through the proteins, and the thickness of the arrows corresponds to the rate of proton transport through each system.

As has been shown previously for UCP2 (Brand et al., 2005; Lee et al., 2015), ANT1, P_i_T-B and UCP4 formed α-helical conformations in OG detergent and retained their overall helical conformations when reconstituted into lipid environments (Figures 2, 4). UCP2 and UCP4 spontaneously self-associated to form tetramers in lipid membranes (Figures 2, 9a), as was observed previously (Ardalan et al., 2021a; Tabefam et al., 2024). These homotetramers were functional in transporting protons across the lipid bilayers - a general characteristic of all UCPs (Brand et al., 2005; Hoang et al., 2012; Orosz and Garlid, 1993; Kreiter et al., 2021). For both UCPs, proton transport activity was higher in the presence of added CL (Figures 5a,b). CD analysis indicated that the inclusion of CL in the membrane may lead to a more relaxed helical conformation of the monomeric subunits of UCP4 (and UCP2) within the homotetramers (Figure 4a). This suggests that conformationally flexible UCP tetramers could facilitate proton translocation (Figures 4a, 5). On the other hand, neither P_i_T nor ANT1 (both of which have comparable three-dimensional structures to UCPs) formed tetramers when reconstituted into lipid membranes (Figures 2, 9a). This finding is consistent with previous studies (Krämer, 1996; Ardalan et al., 2021a; Tabefam et al., 2024). P_i_T monomers exhibited a basal level of proton transport, which increased significantly in the presence of CL. Unlike UCP4, this increase in the proton transport rate, in the presence of CL, could not be attributed to a more relaxed helical structural arrangement in a tetramer. A minor conformational reorganization likely plays a key role in increasing the proton transport activity of dominantly monomeric P_i_T (Figures 2, 4b, 5). Reconstituted ANT1 in EYPC lipid membranes also exhibited proton transport activity both as a monomer (with CL) and as a mixture of monomers and dimers (without added CL) (Figure 2). The presence of CL in the membrane could modify the conformation of ANT1 monomers (CD spectra in Figure 4c), thereby enhancing proton transport function. This observation is consistent with previous studies suggesting that CL acts as a “grease” at the dynamic interface of monomeric units (Kreiter et al., 2021; Beck et al., 2007). However, one of these earlier reports indicated that CL stabilizes interactions of ANT with itself (self-association) and possibly other partners (Claypool, 2009), whereas the other proposes that CL could allow for the close packing of ANT monomers by preventing interactions with other proteins (Ruprecht et al., 2014). A more general conclusion has been suggested in a recent review suggesting that CL is required to accommodate the close association of proteins (including self-association) in the highly crowded and protein-rich environment of the MIM (Schlame, 2021).

### 4.2 Mechanism of proton transport by monomeric subunits

For all 4 MC proteins tested, proton transport occurred only in the presence of long-chain free FAs (Figures 5a–d). Four mechanistic models have been suggested to explain the protonophoric activity of the monomeric form of these carriers in the presence of FAs: (i) the FA cycling model (Engstová et al., 2001; Kreiter et al., 2021; Pohl et al., 2025); (ii) the buffering/cofactor model (Winkler and Klingenberg, 1994); (iii) the long-chain FA shuttling model (Fedorenko et al., 2012); and (iv) the induced transition fit (ITF) model (Bertholet and Kirichok, 2017). In the FA cycling model, protons are transported from the IMS into the matrix by a quick diffusion and flip-flop of protonated FA across the MIM. Meanwhile, the passage of the anionic form of the FA (FA^−^) back to the IMS is supported by MCs, such as ANT, P_i_T and UCPs (Engstová et al., 2001; Kreiter et al., 2021; Beck et al., 2007; Pohl et al., 2025; Garlid et al., 1996; Garlid et al., 1998; Berardi and Chou, 2014; Kreiter et al., 2023). The buffering/cofactor model, originally proposed for UCP1 (Winkler and Klingenberg, 1994), suggests that the deprotonated FA (FA^−^) is bound to the translocon-like channel of the carrier, facilitating proton translocation via their carboxylate group to buffering amino acids of the carrier through the center of the protein. The shuttling model proposes that hydrophobic tails of long-chain FAs are lodged within the carrier, establishing hydrophobic interactions with the protein, and the carboxylate head group then moves through the protein’s cavity, shuttling back and forth between the IMS and matrix. In this model, the head group of the FA becomes protonated in the IMS and deprotonated in the matrix while the FA hydrophobic tail remains bound to the protein (Fedorenko et al., 2012; Bertholet and Kirichok, 2017). A modified version of the shuttling model is called the ITF model (proposed for UCPs) in which FAs induce alternating conformational change in the protein. In this model, as the polar carboxylate head group of a FA moves across the MIM, the UCPs can alternate between the two conformational states in which the protein cavity is either open to the IMS side (c-state) or the matrix side (m-state) of the membrane (Winkler and Klingenberg, 1994; Bertholet and Kirichok, 2017). This so-called alternating access mechanism has been suggested to be a common mechanism of transport in response to substrate binding for all MC protein family members (Ruprecht and Kunji, 2020).

The mechanism by which long-chain FAs facilitate UCP2- and UCP4-mediated proton transport can be qualitatively explained by a recently proposed biphasic two-state model for proton transport by tetrameric UCPs (Ardalan et al., 2021b). In this model, a tetramer is composed of two stable dimeric units, with the monomers within each dimeric unit being in a similar conformational state that is opposite to that of both monomers in the second dimeric unit. That is, the model proposes that the monomeric subunits are functionally and conformationally correlated within a dimer. In the context of the biphasic two-state model, the dependence of UCP2 and UCP4 proton transport activity on long-chain FAs can be generally explained by a combination of translocon-like channel and ITF mechanisms. Accordingly, the monomeric subunits of UCP tetramers act as a H^+^ uniporter and the long hydrocarbon chain becomes anchored to the hydrophobic region of each monomer (four FAs are required per tetramer) by hydrophobic interactions, acting as a cofactor, and the carboxylate head group can move back and forth across the protein as it changes from the c-to m-state and *vice versa*. This oscillating movement of the FA within the protein opening allows protons to be transported by the exchange between UCP-bound FAs and the buffering amino acids of the protein in their path to the matrix side via a translocon-like mechanism, similar to the proton-buffering model described previously (Krauss et al., 2005). It should be also considered that only one of the dimeric units of the tetrameric protein can transport protons at a time, in accordance with the proton gradient across the membrane. In this model the monomeric subunits mutually impact one another and induce interconnected conformational changes. One dimeric unit starts in the c-state and changes to the m-state as the protonated head groups of FAs move from the IMS toward the matrix. Concurrently, the second dimeric unit starts in the m-state and changes to the c-state upon the movement of FA head groups toward the IMS. The stronger hydrophobic interactions provided by the longer, unsaturated hydrocarbon chain of OA enable it to remain bound and participate in subsequent transport cycles (Ardalan et al., 2021b).

The low level of long-chain FA-induced H^+^ translocation mediated by P_i_T and ANT1 might be better explained by the FA cycling model (Engstová et al., 2001; Kreiter et al., 2021; Pohl et al., 2025; Kreiter et al., 2023). This model assumes that the negatively charged FA anion (FA^−^) is attracted to the positively charged surface of the protein and translocates along the lipid-protein interface. In this process, the FA carboxylic group remains in close contact with the positively charged amino acids on the protein surface. Longer and more unsaturated FAs diffuse faster through the lipid bilayer (Kreiter et al., 2021; Pohl et al., 2025; Kreiter et al., 2023).

The inhibitory effects of ATP and GTP on proton transport of UCP2, UCP4, ANT1 and P_i_T carriers differed. It is possible that the differences can be attributed to both the different modes of proton transport and the distinct substrates that these proteins interact with. In ANT1, the nucleotide substrate binding site has a high specificity for adenine nucleotides (ATP in this study) and its c-state inhibitor, CATR, whereas the binding sites of UCP2 and UCP4 accept both ATP and GTP, with a higher affinity for GTP and no affinity for CATR. Conversely, proton transport by P_i_T is not affected by either ATP or GTP (Figures 5, 8; Supplementary Tables S1, S2, S6).

The inhibitory effects of ATP and CATR on ANT1 can be explained by previous molecular dynamics simulation studies, which describe a positive electrostatic potential patch at the protein-lipid interface (Ardalan et al., 2021b). When ATP and/or CATR (both of which carry a charge of −4) bind to the positively charged substrate binding site of ANT1, the resulting local conformational change reduces this electrostatic potential. Consequently, the attraction of anionic FAs (as proton transport activators) to the ANT1 surface is reduced, leading to reduced (or inhibited) proton transport. However, when GTP binds to ANT1, it adopts a different orientation that has less impact on ANT1’s electrostatic potential (Kreiter et al., 2021). The surface electric charge of ANT1 is directly correlated with the inhibition potency of purine nucleotides and is supported by observed competition between purine nucleotides and FAs for the binding site in ANT1 (Kreiter et al., 2021; Kreiter et al., 2023).

Our results for UCP2 and UCP4 are consistent with previous studies showing that purine nucleotides bind to the proteins in their c-state, near the center of the central cavity, occluding the UCP translocation pathway (Ardalan et al., 2021b; Bertholet and Kirichok, 2017). This binding likely obstructs (or reduces the rate of) the movement of FA headgroups between the IMS and the matrix, thereby preventing the protein’s conformational change or locking it in an intermediate state (Ardalan et al., 2021b; Jones et al., 2023). Moreover, inhibition by ATP and GTP could also be the consequence of the neutralization of the positive charges of arginine triplet residues (e.g., R88, R185, and R279 in UCP2) (Bertholet and Kirichok, 2017) through interactions with the negatively charged phosphate groups. These arginine triplet residues are suggested to be the substrate contact points in MCs and have been suggested to attract FAs toward the protein cavity to initiate proton transport (Ardalan et al., 2021b). Additionally, previous reports have suggested that ATP and long-chain FA anions might compete to bind near or within the proton translocation pathway of UCP1 (Fedorenko et al., 2012; Bertholet and Kirichok, 2017). It was also proposed that long-chain FAs could potentially counteract the inhibitory effects of ATP on UCP1 (Fedorenko et al., 2012). Given the substantial structural differences between FA anions and ATP, their binding sites on UCP1 are unlikely to be identical. However, these sites may partially overlap or be located in close proximity, leading to electrostatic repulsion and competition between the two negatively charged species (Bertholet and Kirichok, 2017). Alternatively, binding either of ATP or FA may discourage the binding of the other through negative allosteric effects. Our findings support this hypothesis for UCP2, as ATP was less effective as an inhibitor of proton transport in the presence of OA than in the presence of PA. This was also the case for the inhibition of UCP2 by GTP. However, in the case of UCP4, ATP and GTP were more effective at inhibiting proton transport in the presence of the longer chain OA, which could be the result of stronger interaction of ATP and GTP with UCP4.

### 4.3 Proton transport by heterotetrameric complexes

Only bands corresponding to tetrameric complexes were visible on semi-native gels when UCP4, ANT1 and P_i_T were reconstituted into lipid membranes together in different stoichiometric ratios, indicating that no proteins remained monomeric. Since ANT1 and P_i_T are not capable of forming homotetramers, all combinations of these MC proteins must have resulted in the formation of heterotetramers (Figures 3, 9b). The current study reveals, for the first time, the possibility of the cooperative interactions and joint functional roles of these MC proteins in mitochondrial proton transport regulation. In our experimental system, the basal proton transport rates for UCP2 and UCP4 homotetramers were determined to be 3.5 ± 0.1 and 2.6 ± 0.06, respectively (Figures 5a, b; Supplementary Table S1). However, when combined in a 1:1 stoichiometric ratio to form heterotetramers the resulting proton transport rate (3.4 ± 0.3) was close to the rate of UCP2 on its own, suggesting that their combined function does not result in an additive effect. This indicates that, although UCP2 and UCP4 may cooperate when combined, their proton transport activity is not significantly enhanced or diminished compared to UCP2 alone (Figures 6, 9b; Supplementary Table S3). The similar inhibition by ATP (52.9%) (Figure 6; Supplementary Table S3) for the 1:1 (UCP2:UCP4) combination, which closely aligns with the inhibition observed for individual UCP2 (55.1%) (Figure 5a; Supplementary Table S1), further supports the idea that the proton transport activity of the heterotetramers remains comparable to that of the individual UCP2.

The significantly enhanced proton transport rate for the 1:1 stoichiometric combination of ANT1 with UCP2 (13.90 ± 0.70) (Figures 6, 9b; Supplementary Table S3), compared to individual ANT1 (0.50 ± 0.30) and UCP2 (3.50 ± 0.10) proteins (Figures 5, 9a; Supplementary Table S1), is suggestive of a strong cooperativity between the proteins in the heterotetramers. This cooperativity could possibly be explained by ANT’s role in mitochondrial energy exchange complementing UCP2’s transport capabilities.

For the UCP2:P_i_T combination (1:1 stoichiometry), the transport rate (7.60 ± 0.40) (Figures 6, 9b; Supplementary Table S3) was significantly higher (approximately two times higher) than UCP2 on its own (3.5 ± 0.1), and much higher than P_i_T on its own (0.46 ± 0.30) (Figures 5, 9a; Supplementary Table S1). That ATP did not significantly inhibit the UCP2:P_i_T combination (30.3% inhibition) compared to other combinations, may indicate a lower affinity for ATP of this hetero-oligomeric complex (Figure 6; Supplementary Table S3).

Proton transport by the combination of UCP4 and ANT1 (1:1) (7.80 ± 0.40) (Figures 6, 9b; Supplementary Table S3) was significantly (three times) higher than UCP4 (2.57 ± 0.06) on its own and much higher than ANT1 alone (0.50 ± 0.30) (Figures 5, 9a; Supplementary Table S1), suggesting positive cooperativity between these 2 MCs. The significant inhibition by ATP of proton transport by the 1:1 combination of UCP4 and ANT1 (75.6%) (Figure 6; Supplementary Table S3) indicates that the UCP4-ANT1 heterotetramer may be regulated similarly to UCP2-ANT1 heterotetramers (79.1% inhibition by ATP) (Figure 6; Supplementary Table S3). That heterotetramers including UCPs and ANT1 respond similarly to ATP supports the idea that ANT1 cooperatively enhances the proton transport activity of UCPs, and that ATP modulates this cooperative interaction effectively. However, the proton transport rate of heterotetramers formed by the 1:1 combination of UCP4 and P_i_T (2.10 ± 0.10) (Figures 6, 9b; Supplementary Table S3) was not significantly enhanced compared to UCP4 alone, showing that the positive cooperativity observed for other combinations is not a universal effect of any combination of MCs, but is specific to certain combinations (UCP2-ANT1 and UCP4-ANT1 in this instance).

Finally, the significantly higher proton transport rate by ANT1 combined with P_i_T (6.0 ± 0.4), illustrates the possibility of strong cooperative interaction among MCs (Figures 6, 9b; Supplementary Table S3) other than UCPs. The significant inhibition by ATP (55.8%) reflects the cooperative yet possibly distinct regulatory mechanism of the ANT1- P_i_T pair (Figure 6; Supplementary Table S3). This observation suggests that while neither ANT1 nor P_i_T are effective proton transporters on their own, their combination can significantly enhance their proton transport activity and that this function can be modulated by ATP.

The existence of only heterotetrameric complexes of different MCs is suggestive of a tendency for heterotypic protein-protein interactions and regulatory mechanisms in mitochondrial proton transport.

### 4.4 Mechanism of proton transport by heterotetrameric protein complexes

A simple mechanism of proton transport by monomers within heterotetrameric complexes can also be explained by the two-state model (Ardalan et al., 2021b). The organization of monomeric subunits within heterotetramers varies according to the stoichiometric ratio of reconstituted proteins. Assuming the likelihood that homo- and heterotypic interactions are similar, when a 1:1 ratio of 2 MCs is used, the formation of two different homodimeric units is highly possible. In a heterotetramer with a 3:1 or 1:3 ratio of two different MC proteins, the resulting complexes are likely to contain one homodimer and one heterodimer. Furthermore, the proton transport activity is significantly different for individual proteins and protein combinations. Tetramers formed from 3:1 (and 1:3) combinations may consist of a mixture of homo- and heterodimers.

**FIGURE 10:**
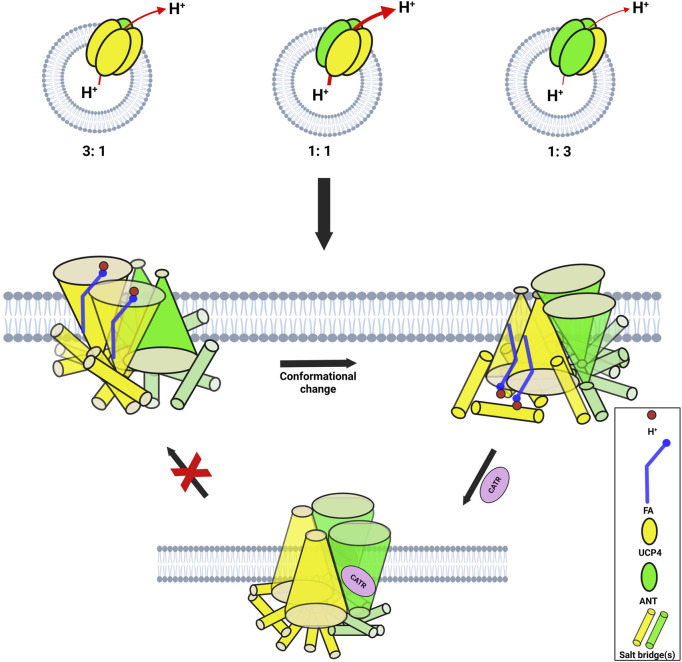
Mechanism of proton transport and inhibition in a 1:1 UCP4:ANT1 heterotetrametric complex. The ANT1 dimers (green) cooperatively enhance the proton transport function of the UCP4 homodimers (yellow), which remain as the primary functional units. Inhibition of ANT1 by CATR blocks the alternating access mechanism, thereby preventing proton transport. The cylinders represent the functional state (not the precise location) of salt bridges that can switch between open and closed states.

Analysis of 3:1 and 1:3 stoichiometric combinations of MCs is performed on the UCP4:ANT1 system. The 1:1 combination of the lesser-studied UCP4 can be compared to the 1:1 combination of UCP2 and ANT1 (Figures 5, 8). This equimolar UCP4-ANT1 tetramer most likely comprises a combination of a homodimer of UCP4 and a homodimer of ANT1. When reconstituted in a 3:1 ratio (UCP4:ANT1), the most likely (or predicted to be most abundant) structure of the heterotetramers is a UCP4 homodimer combined with a UCP4-ANT1 heterodimer. Conversely, in a 1:3 ratio (UCP4: ANT1), the most probable heterotetramer would contain a homodimer of ANT and a UCP4-ANT1 heterodimer (Figure 10). According to the biphasic transport model, proton transport occurs through an alternating mechanism involving dimeric units. During one phase, the UCP4 homodimers are in the c-state and accept protons while the ANT1 homodimers are in the m-state. In the next phase, the UCP4 homodimers switch to the m-state and release protons to the matrix as the ANT1 homodimers switch to the c-state. Although there is a possibility that the dimeric ANT1 units can also cause FA^−^ cycling, it can be assumed that most of the proton transport occurs through the UCP4 homodimers, with the ANT1 dimers enhancing this function (Figure 10). The importance of ANT in the 1:1 combination was further verified using CATR, an inhibitor of ANT’s c-state (Pebay-Peyroula et al., 2003). CATR tightly blocks ANT1 from switching between conformations, thus preventing alternating access; when part of a heterotetramer with UCP, preventing conformational switching of ANT1 between the m- and c-state prevents the entire tetrameric complex from engaging in alternating access. Consequently, the transport rate significantly decreases, approaching the activity level of individual UCP4 function (Figures 8–10; Supplementary Tables S7, S8). We also studied the inhibitory effect of CATR on co-reconstituted UCP2 and ANT1 (1:1 ratio). Comparable to the UCP4-ANT1 pair, blocking ANT1 in the UCP2:ANT1 heterotetramer with CATR prevents alternating access of the entire tetramer, leading to a drastic decrease in proton transport.

In summary, we propose that the proton transport mechanism by heterotetrameric complexes of MCs is influenced by the stoichiometric ratio of the proteins involved. Different combinations, particularly the 1:1 and 3:1 (and 1:3) ratios of UCP4 and ANT1, result in specific structural arrangements of homodimers and heterodimers within the tetramers, leading to distinct functional properties compared to individual proteins. For example, in the 1:1 (UCP4:ANT1) combination the higher proton transport rates can be explained by the cooperative role of ANT1 dimers in enhancing the transport functionality of UCP4 dimers. The critical role of ANT1 in this process is underscored by the significant decrease in transport rate when conformational switching by ANT is inhibited in UCP4:ANT1 and UCP2:ANT1 (1:1) combinations, implying the complex interplay between these MC proteins in facilitating efficient proton transport.

### 4.5 Conclusion

In this study, we explored the functional dynamics of a group of MC proteins involved in regulation of the proton motive force and ATP synthesis in mitochondria (two UCPs, ANT1, and P_i_T) in an effort to elucidate their individual and combined roles in proton transport across the MIM. Our findings provide compelling evidence that these proteins not only function independently but can also form heterotetrameric complexes that exhibit distinct and enhanced proton transport activities as compared to individual proteins.

We also demonstrated that UCP2 and UCP4 form functional tetramers that facilitate proton transport, with their activity modulated by CL and long-chain FAs. Concomitantly, ANT1 and P_i_T-B, while not forming tetramers on their own, showed a similar dependence on interactions with long-chain FAs for proton transport, supporting the idea that these interactions are crucial for their function. Our experiments with the 1:1 combination of UCP2-ANT1 and UCP4-ANT1 revealed a cooperative effect that led to increased proton transport rates compared to the individual proteins. Such cooperative behavior may be a general feature of UCP interactions with ANT1. Functional synergy appears to be fine-regulated by inhibitors ATP, GTP, and CATR, suggesting a broader relevance to mitochondrial bioenergetics. ANT1 could therefore have a critical role in regulating the proton transport function of UCPs in the heterotetrameric complexes. Generally, the importance of stoichiometry of proteins in determining the functional behaviour of heterotetramers is emphasized.

Finally, it is important to recognize that our biophysical experiments may provide valuable insights but do not fully capture the complexities of *in vivo* environments. The physiological relevance and behavior of these molecular interactions in living systems remain to be clarified. Validation of these findings *in vivo* will help to confirm the physiological relevance of our observations and will also reveal differences in behavior and function under more complex cellular conditions. In the scope of mitochondrial molecular physiology, we hope that the emphasis of this study on intricate functional relationships between these MC proteins that affect efficiency of mitochondrial proton transport can open new avenues for understanding mitochondrial bioenergetics, and the potential for targeting MC protein interactions in therapeutic strategies.

## Data Availability

The original contributions presented in the study are included in the article/Supplementary Material, further inquiries can be directed to the corresponding authors.
