# Supplementary material for: Interconnected roles of mitochondrial carrier proteins ANT, PiT, and UCPs in proton transport
Source: Front Mol Biosci. 2025 Nov 24;12:1650261. doi: 10.3389/fmolb.2025.1650261 (PMC12682859; doi:10.3389/fmolb.2025.1650261)
Supplement: Supplementary file 1 [file Supplementaryfile1.pdf]

Supplementary Material

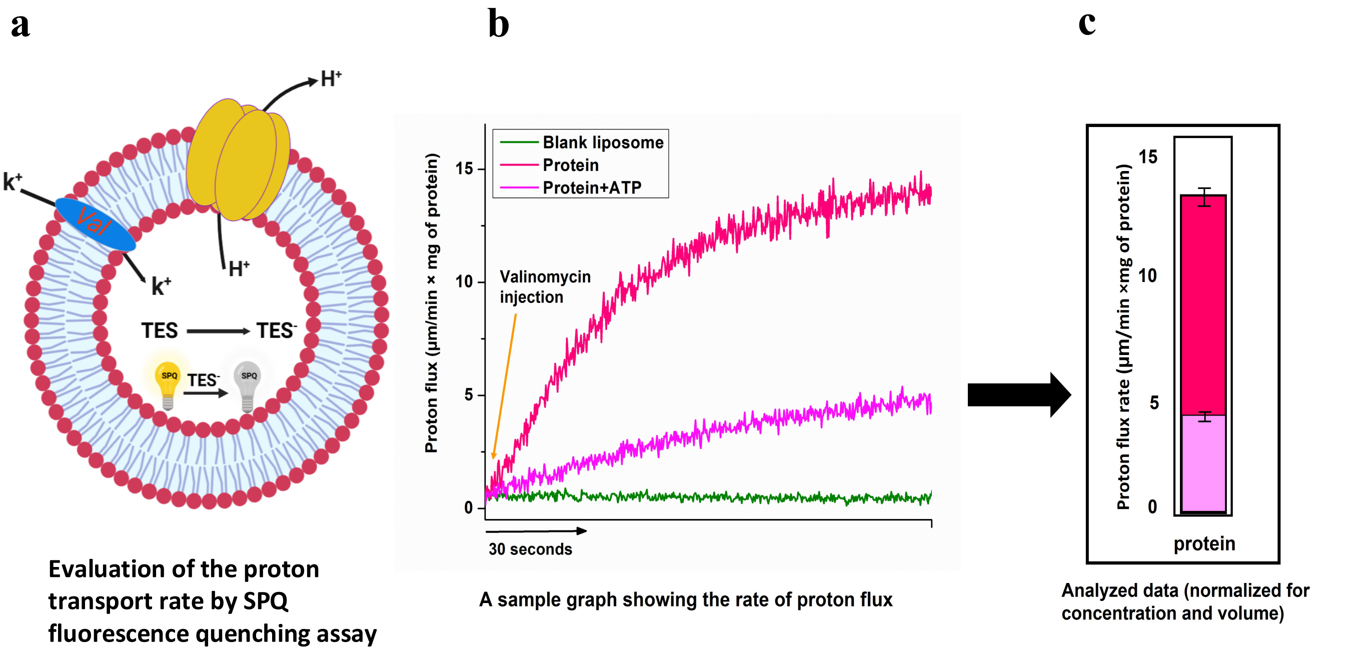

Figure S1. Schematic of proton transport assay and data analysis.
(a) A diagrammtic representation of a liposome with a reconstituted protein of interest is shown in TES buffer for measuring proton transport upon addition of valinomycin (and free fatty acid, not shown) using the SPQ fluorescence quenching assay. (b) Representative graph of proton efflux rate over time using the fluorescence quenching assay under three conditions: blank liposomes (green), protein without ATP (pink), and protein with ATP (magenta). (c) Quantified proton flux rate (µmol/min/mg protein) for the liposomes containing protein without (pink) and with (magenta) added ATP, after subtraction of proton leak from blank liposomes shown in the graph in (b), with error bars showing standard deviation.

Table S1. Total proton transport rates for UCP2, UCP4, ANT1, and P_iT reconstituted in EYPC lipid vesicles without CL. Proton transport was determined in the presence of palmitic acid (PA) or oleic acid (OA) as free fatty acid (FFA) proton transport activators, and in the absence and presence of purine nucleotides ATP or GTP. Refer to Materials and Methods for experimental conditions including the protein amounts, and concentrations of FFA and nucleotides. Proton transport rates are reported in µmol of proton/min/mg protein.

Protein	FFA	Purine Nucleotide	H^+ Transport Rate	Inhibition%
UCP2	PA	-	3.5 ± 0.1	-
		ATP	1.57 ±0.04	55.1
		GTP	0.8 ± 0.01	77.1
	OA	-	4.48 ± 0.1	-
		ATP	3.07 ± 0.03	31.5
		GTP	1.92± 0.13	57.1
UCP4	PA	-	2.57 ± 0.06	-
		ATP	1.22 ±0.03	52.5
		GTP	0.57 ± 0.03	77.8
	OA	-	3.71 ± 0.07	-
		ATP	0.92 ± 0.06	75.2
		GTP	0.4± 0.02	89.2
ANT1	PA	-	0.5 ±0.03	-
		ATP	0.27 ± 0.02	46
		GTP	0.40 ± 0.09	20
	OA	-	1.5 ± 0.2	-
		ATP	0.15 ± 0.04	90
		GTP	1.2 ± 0.2	20
P_iT	PA	-	0.46 ± 0.3	-
		ATP	0.2 ± 0.15	56.5
		GTP	0.26 ± 0.09	43
	OA	-	1.10 ±0.05	-
		ATP	0.88 ± 0.09	20
		GTP	0.96 ± 0.02	12.7

Table S2. Total proton transport rates for UCP2, UCP4, ANT1, and P_iT reconstituted in EYPC lipid vesicles with 2.5% CL added. Proton transport was determined in the presence of palmitic acid (PA) or oleic acid (OA) as free fatty acid (FFA) proton transport activators, and in the absence and presence of purine nucleotides ATP or GTP. Refer to Materials and Methods for experimental conditions, including protein amounts, and concentrations of FFA and nucleotides. Proton transport rates are reported in µmol of proton/min/mg protein.

Protein	FFA	Purine Nucleotide	H^+ Transport Rate	Inhibition%
UCP2	PA	-	5.16 ± 0.24	-
		ATP	2.55 ± 0.09	50.6
		GTP	1.58 ± 0.08	69.4
	OA	-	6.4 ± 0.05	-
		ATP	2.78 ± 0.3	56.7
		GTP	2.98 ± 0.2	53.4
UCP4	PA	-	3.29 ± 0.17	-
		ATP	0.5 ± 0.07	84.8
		GTP	0.42 ± 0.09	87.2
	OA	-	4.71 ± 0.4	-
		ATP	1.65 ± 0.06	64.9
		GTP	1.15 ± 0.02	75.6
ANT1	PA	-	1.27 ± 0.17	-
		ATP	0.3± 0.07	76.4
		GTP	0.96 ± 0.16	24.4
	OA	-	2.8 ± 0.09	-
		ATP	0.3 ± 0.04	89.3
		GTP	2.45 ± 0.2	12.5
P_iT	PA	-	2.35 ± 0.16	-
		ATP	1.88± 0.04	20
		GTP	1.65 ± 0.08	29.8
	OA	-	3.79 ± 0.2	-
		ATP	3.2 ± 0.1	15.6
		GTP	3.44 ± 0.04	9.2

Table S3. Total proton transport rates of combinations of UCP2, UCP4, ANT1 and PiT reconstituted at a 1:1 stoichiometric ratio in EYPC lipid vesicles without added CL. Transport was activated using palmitic acid (PA) and was measured in the absence or presence of ATP. Refer to Materials and Methods for the experimental conditions, including protein amounts, and concentrations of PA and ATP. Transport is reported in µmol proton/min/mg protein. Inhibition of transport in the presence of ATP as compared to in the absence of ATP is reported as a percentage.

Protein Combination (1:1)	ATP	H^+ Transport Rate	% Inhibition
UCP2: UCP4	-	3.4 ± 0.3	
	+	1.6 ± 0.2	52.9
UCP2: ANT1	-	13.9 ± 0.7	
	+	2.9 ± 0.14	79.1
UCP2: P_iT	-	7.6 ± 0.4	
	+	5.3 ± 0.7	30.3
UCP4: ANT1	-	7.8 ± 0.4	
	+	1.9 ± 0.08	75.6
UCP4: P_iT	-	2.1 ± 0.1	
	+	1.9 ± 0.2	9.5
ANT1: P_iT	-	6.02 ± 0.4	
	+	2.66 ± 0.4	55.8

Table S4. Total proton transport rates for UCP4:ANT1, 1:1, 3:1 and 1:3 stoichiometric combinations reconstituted in EYPC lipid vesicles without added CL. Proton transport was determined in the presence of palmitic acid (PA) or oleic acid (OA) as free fatty acid (FFA) proton transport activators, and in the absence and presence of purine nucleotides ATP or GTP. Refer to Materials and Methods for experimental conditions, including FFA and nucleotide concentrations. Proton transport rates are reported in μmol of proton/min/mg protein. Data for the 1:1 ratio (PA, no CL, ±ATP) are reproduced from Table S3.

Ratio	FFA	Purine Nucleotide	H+ Transport Rate	Inhibition %
1:1	PA	-	7.8 ± 0.41	
		ATP	1.9 ± 0.08	75.6
		GTP	1.28 ± 0.12	83.6
	OA	-	11.0 ± 0.15	
		ATP	3.7 ± 0.9	66.4
		GTP	3.5 ± 0.6	68.2
3:1	PA	-	3.8 ± 0.07	
		ATP	2.1 ± 0.06	44.7
		GTP	0.2 ± 0.1	94.8
	OA	-	7.2 ± 0.02	
		ATP	3.8 ± 0.1	47.2
		GTP	1.2 ± 0.6	83.3
1:3	PA	-	1.97 ± 0.16	
		ATP	0.8 ± 0.1	59.4
		GTP	0.6 ± 0.2	69.5
	OA	-	4.05 ± 0.6	
		ATP	0.5 ± 0.6	87.6
		GTP	2.3 ± 0.1	43.2

Table S5. Total proton transport rates for UCP4:ANT1, 1:1, 3:1 and 1:3 stoichiometric combinations reconstituted in EYPC lipid vesicles containing 2.5% added CL. Proton transport was determined in the presence of palmitic acid (PA) or oleic acid (OA) as the free fatty acid (FFA) proton transport activators, and in the absence or presence of purine nucleotides ATP or GTP. Refer to Materials and Methods for experimental procedures, including the protein amounts, and concentrations of FFA and nucleotides. Proton transport rates are reported in μmol of proton/min/mg protein.

Ratio	FFA	Purine Nucleotide	H^+ Transport Rate	Inhibition%
1:1	PA	-	11.3 ± 0.9	
		ATP	4.05 ± 0.1	64.2
		GTP	2.3 ± 0.2	79.6
	OA	-	14.1 ± 0.7	
		ATP	3.3 ± 0.2	76.6
		GTP	1.85 ± 0.07	86.9
3:1	PA	-	5.3 ± 0.3	
		ATP	2.1 ± 0.3	60.4
		GTP	0.45 ± 0.2	91.5
	OA	-	7.5 ± 0.4	
		ATP	3.7 ± 0.1	50.1
		GTP	1.2 ± 0.14	84
1:3	PA	-	2.5 ± 0.4	
		ATP	1.0 ± 0.1	60
		GTP	1.07 ± 0.2	57.2
	OA	-	3.3 ± 0.3	
		ATP	1.4 ± 0.3	57.6
		GTP	1.02 ± 0.1	69.1

Table S6. Comparison between total proton transport and CATR inhibited proton transport for UCP2, UCP4, ANT1, UCP4:ANT1 (1:1) and UCP2:ANT1 (1:1) in EYPC lipid vesicles containing 2.5% added CL. Proton transport was determined in the presence of palmitic acid (PA) as proton transport activator. Refer to Materials and Methods for experimental procedures, including PA and CATR concentrations. All the units are in μmol of proton/min/mg protein. The total proton transport data for UCP4, UCP2, and ANT1 are reproduced from Table S2.

**Protein (Combination)**	**CATR**	**H^+ Transport Rate**	**% Inhibition**
UCP2	-	5.16 ± 0.24	
	+	5.07 ± 0.3	1.7
UCP4	-	3.29 ± 0.17	
	+	3.2 ± 0.09	2.7
ANT1	-	1.27 ± 0.17	
	+	0.38 ± 0.05	70.1
UCP2: ANT1 (1:1)	-	16.3 ± 0.3	
	+	4.3 ± 0.2	73.6
UCP4:ANT1 (1:1)	-	11.3 ± 0.9	
	+	3.32 ± 0.2	71
